# Targeted metagenomics using probe capture detect a larger diversity of nitrogen and methane cycling genes in complex microbial communities than traditional metagenomics

**DOI:** 10.1093/ismeco/ycaf183

**Published:** 2025-11-01

**Authors:** Henri M P Siljanen, Lokeshwaran Manoharan, Angus S Hilts, Alexandre Bagnoud, Ricardo J E Alves, Christopher M Jones, Melina Kerou, Felipa L Sousa, Sara Hallin, Christina Biasi, Christa Schleper

**Affiliations:** Department of Environmental and Biological Sciences, University of Eastern Finland, Kuopio FI-70210, Finland; Department of Functional and Evolutionary Ecology, University of Vienna, Vienna A-1030, Austria; Department of Functional and Evolutionary Ecology, University of Vienna, Vienna A-1030, Austria; National Bioinformatics Infrastructure Sweden (NBIS), SciLifeLab, Department of Laboratory Medicine, Lund University, 223 81 Lund, Sweden; Department of Functional and Evolutionary Ecology, University of Vienna, Vienna A-1030, Austria; Department of Functional and Evolutionary Ecology, University of Vienna, Vienna A-1030, Austria; Membratec S, Ecoprac de Daval C 1, CH-3960 Sierre, Switzerland; Department of Functional and Evolutionary Ecology, University of Vienna, Vienna A-1030, Austria; Department of Forest Mycology and Plant Pathology, Swedish University of Agricultural Sciences, 750 07 Uppsala, Sweden; Department of Functional and Evolutionary Ecology, University of Vienna, Vienna A-1030, Austria; Department of Functional and Evolutionary Ecology, University of Vienna, Vienna A-1030, Austria; Department of Forest Mycology and Plant Pathology, Swedish University of Agricultural Sciences, 750 07 Uppsala, Sweden; Department of Environmental and Biological Sciences, University of Eastern Finland, Kuopio FI-70210, Finland; Department of Ecology, University of Innsbruck, Innsbruck A-6020, Austria; Department of Functional and Evolutionary Ecology, University of Vienna, Vienna A-1030, Austria

**Keywords:** metagenomics, nitrogen cycling, methane cycling, probe hybridization targeted metagenomics, PCR amplicon sequencing, shotgun metagenomics

## Abstract

Microorganisms are key players in the global cycling of nitrogen and carbon, controlling their availability and fluxes, including the emissions of the powerful greenhouse gases nitrous oxide and methane. Standard sequencing methods often reveal only a limited fraction of their diversity, because of their low relative abundance, the insufficient sequencing depth of traditional metagenomes of complex communities, and limitations in coverage of DNA amplification-based assays. Here, we developed and tested a targeted metagenomics approach based on probe capture and hybridization to simultaneously characterize the diversity of multiple key metabolic genes involved in inorganic nitrogen and methane cycling. We designed comprehensive probe libraries for each of the 14 target marker genes comprising 264 111 unique probes. In validation experiments with mock communities, targeted metagenomics yielded gene profiles similar to the original communities. Only GC content had a small effect on probe efficiency, as low GC targets were less efficiently detected than those with high GC, within the mock communities. Furthermore, the relative abundances of the marker genes obtained using targeted or traditional shotgun metagenomics were significantly correlated. In addition, using archaeal *amoA* genes as a case-study, targeted metagenomics identified a substantially higher taxonomic diversity and a larger number of sequence reads per sample, yielding diversity estimates 28 or 1.24 times higher than shotgun metagenomics or amplicon sequencing, respectively. Our results show that targeted metagenomics complements current approaches to characterize key microbial populations and functional guilds in biogeochemical cycles in different ecosystems, enabling more detailed, simultaneous characterization of multiple functional genes.

## Introduction

The global nitrogen (N) and carbon (C) cycles are essential processes of the Earth’s biosphere and crucial for ecosystem functioning [1–5]. All major N transformation processes (i.e. nitrogen fixation, nitrification, denitrification, dissimilatory nitrate reduction to ammonium, and anaerobic ammonium oxidation, or anammox) are performed exclusively by the functional guilds of bacteria, archaea, and eukaryotes [6–9]. Their activities regulate N availability for primary producers and microorganisms across ecosystems and control the production and consumption of the potent greenhouse gas nitrous oxide (N_2_O) and other gaseous N compounds through processes such as nitrification, denitrification, and non-denitrifier N_2_O reduction [10–13]. Methane (CH_4_) is another major product of the microbial trophic chain underlying C cycling, and, like the most inorganic N compounds, its production and consumption are also regulated by highly specific groups of microorganisms [14–16]. Importantly, CH_4_ is the second most potent greenhouse gas after carbon dioxide and together with N_2_O, contributes at least 25% of the total global warming caused by greenhouse gases [17–19].

Despite their important ecological role, microorganisms participating in inorganic N and CH_4_ transformations typically constitute a small fraction of microbial communities in most soil, sediment, and aquatic ecosystems [20–24]. Community profiling based on 16S rRNA genes only allows for very limited information about microbial functions [25]. Even in the rare cases where functions can be inferred from organism phylogeny, the striking genetic and functional diversity within functional guilds remains concealed, such as that among their key metabolic enzymes (e.g. ammonia monooxygenase (*amoA*) [26], methyl co-enzyme M reductase (*mcrA*) [27]). Additionally, taxonomy-based approaches are inadequate in elucidating processes that have a broader taxonomic distribution than originally thought (e.g. [27–30]). Shotgun metagenomics is the typical method of choice for obtaining a relatively unbiased picture of the natural microbiome, provided that issues with sample preparation, DNA extraction, library preparation, and sequencing method can be ruled out [30–33]. Nevertheless, in complex and diverse microbial communities such as those in soils and sediments, even deeply-sequenced metagenomes do not uncover the full diversity of microbial functional guilds and key functional genes owing to their typically low relative abundances within the broader community [21, 24, 34, 35]. As a low-cost alternative, the characterization of specific functional groups has long relied on the PCR amplification of genes that encode key metabolic enzymes, as for example, of organisms involved in various N and CH_4_ cycling pathways [26, 36–39]. However, the gene diversity captured by gene-specific PCR assays is limited by the primers used, which introduce biases that make comparisons among different genes or samples difficult [40–43]. Furthermore, in complex microbial communities, target genes of interest are encoded by a large diversity of organisms, and usually include a large fraction of novel gene sequence variants from uncharacterized organisms [12, 29]. Thus the geometric expansion of genomic data has made it increasingly obvious that designing a single PCR primer pair to target the inherent diversity of metabolic genes is highly problematic [44]. Therefore, linking ecosystem functions to microbial communities is often only possible through the complex and resource-intensive combinations of meta-omics and isotope labelling approaches [34, 45].

Targeted high-throughput sequencing approaches, known as probe capture, also called hybridization capture, hybridization-based target enrichment, or captured metagenomics, have been used to study complex eukaryotic samples such as human exons, ancient human genomes, plant transcriptomes, and cancer marker single nucleotide polymorphisms [46–51]. A few studies have also used this approach to facilitate the in-depth study of microbial communities using either 16S rRNA genes or other microbial functional genes [52–55]. The probe capture approach relies on targeting specific short sequences within a broader genomic pool (e.g. full genomes, genome or gene fragments) with biotin-labelled probes. The probes that hybridize with their target regions are then selectively captured from the full genomic library using streptavidin-labelled magnetic beads [56]. Different from sequence capture, where multiple probes are designed to cover a whole exon, captured metagenomics relies on the large databases of multiple target genes clustered at predefined similarity cut-offs, which are used to design a large number of generic probes that are able to capture an extended sequence space for each of these sequence clusters [53, 57].

To improve the detection and characterization of important but low-abundant microbial guilds involved in N and CH_4_ cycling in natural communities, we developed and evaluated a probe-based targeted metagenomics approach to key genes involved in these processes. In addition to the enhanced resolution and coverage of genetic sequence space, this approach allows the parallel analysis of distinct steps within N- and CH_4_- cycling pathways, thus combining the advantages of both shotgun metagenomics and amplicon-based approaches. Here, we present a new probe set for targeting 14 distinct functional genes involved in inorganic N cycling (i.e. N fixation, nitrification, denitrification, anammox, and dissimilatory nitrate reduction to ammonium), and three genes involved in CH_4_ production and consumption. We evaluated our approach using mock communities comprising DNA from microorganisms involved in inorganic N cycling, or CH_4_ production or consumption with varying gene GC mol% content, which is known to affect probe hybridization efficiency. As a proof-of-concept for complex communities, we performed shotgun metagenomics and targeted metagenomics on agricultural and wetland soil samples and compared the diversity of two functional markers, the archaeal *amoA* genes (marker for ammonia oxidizers) and *nosZ* genes (marker for N_2_O reduction).

## Materials and methods

### Construction of the target gene databases

Target gene databases (TDBs) were constructed, containing all identifiable variants for the following key genes: the nitrogenase iron subunit (*nifH*), bacterial and archaeal ammonia monooxygenase subunit A (*amoA)*, nitrite oxidoreductase beta subunit (*nxrB)*, hydrazine oxidoreductase A (*hzoA)*, formate dependent nitrite reductase (*nrfA*), periplasmic nitrate reductase alpha subunit (*napA*), respiratory nitrate reductase alpha subunit (*narG*), copper-containing nitrite reductase (*nirK*), cytochrome cd_1_ nitrite reductase (*nirS*), nitric oxide reductase subunit B (*norB*), N_2_O reductase (*nosZ*), particulate CH_4_ monooxygenase subunit A (*pmoA*), soluble CH_4_ monooxygenase component A alpha chain (*mmoX*), and methyl-coenzyme M reductase I subunit alpha (*mcrA*).

**Figure 1:**
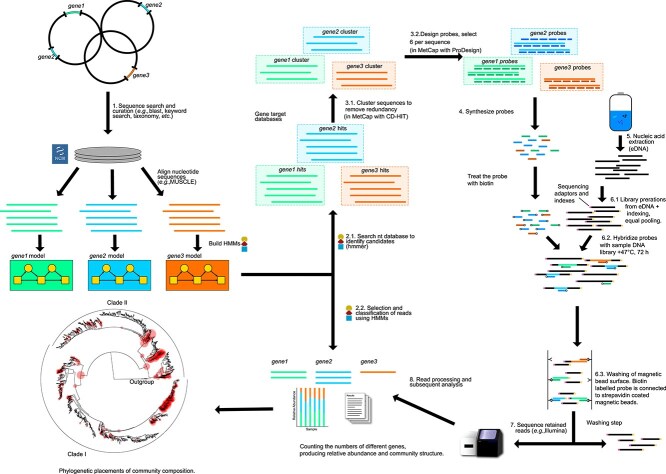
Illustration of the pipeline for the generation of HMM models for each gene, and how these models are used to study community composition with targeted metagenomics: 1. Steps followed for the generation of sequence databases for the genes of interest and subsequent building of the respective HMM models for each gene; 2.1. The produced HMM models were used to recruit more gene variants from the NCBI nt database and generate a target gene database for probe design; 2.2. The same HMM models were also used to classify the reads captured downstream; 3.1. Clustering of the target gene database with CD-HIT (in MetCap pipeline [57]); 3.2. Design and selection of six 50-mer probes for each sequence cluster (in MetCap [57]); 4. Synthesis and biotinylation of the designed probes by Roche; 5. DNA extraction of the sample of interest; 6.1. Sequencing libraries preparation and addition of sequencing adaptors and indexes; 6.2. Hybridization of the probes to indexed DNA libraries for 72 h at +47°C; 6.3. Purification of the DNA hybridized to the probes with streptavidin coated magnetic beads; 7. Sequencing of the hybridized libraries with Illumina MiSeq; 8. Read processing with HMM profiles and taxonomy assignment for the genes of interest.

Hidden Markov model (HMM) models were generated in order to identify all variants of the target genes from public databases (Fig. 1). These models were built based on reference sequence alignments from curated databases already available for selected genes, such as *amoA* [26], *pmoA* [36], *nosZ* [58], *nirK, nirS, nor* [59], and the Fungene repository [60]. For target genes where alignments were not available, reference alignments were generated from gene sequence data publicly available on the NCBI WGS-database, from the full length open-reading frame of each subunit, to cover all known diversity in each gene. Structure-based searches of the Genbank nt- and envnt- database were subsequently performed with nhmmer using the generated HMMs for every target gene [61] on a local supercomputer cluster (Center for Scientific Computing CSC, Espoo, Finland) (July 2017). The HMM models are available through Zenodo [62], in script: probe-capture/2-selected_outputs/hmmer_profiles.zip. This sequence search selection process generated ~600 000 unique sequences across all gene families. The obtained database was clustered to 100% identity in order to remove duplicate sequences in database with cd-hit [63] and inspected to exclude 16S rRNA genes. The final output comprised the TDB; available through Zenodo [62] probe-capture/2-selected_outputs/all_genes_compiled_ncbi_nt_envnt_fungene_split_file_0.fasta.gz -file (four files ..file_0–3.fasta.gz). The workflow can be seen in Fig. 1. Scripts for Target gene database search is available in Zenodo [62] /probe-capture/tree/main/3-other_scripts/Henri_scripts/Script_probe_capture.sh -folder.

### Generation of probes for target genes

The MetCap bioinformatics pipeline was used as a protocol to produce probes from highly complex datasets for targeted metagenomics [57]. Default parameters were used for designing up to six unique 50-mer probes for each sequence cluster in the TDBs (clustered with an 80% identity clustering threshold), with melting temperature of 47°C, resulting in a set of 263 111 unique gene-specific probes (Fig. 1), and in Zenodo [62] probe-capture/2-selected_outputs/Final_N_probes.list1.fasta -file. The probes were synthesized by NimbleGen SeqCap EZ (Roche NimbleGen, Inc., Madison, USA) as Custom design (since the MetCap pipeline was used for probe design, probe order was done with the Roche HyperDesign tool: www.hyperdesign.com), with biotin labelling to enable retrieval of the hybridized targets using streptavidin coated magnetic beads [53, 57].

### Extraction of DNA from cultures and generation of mock community DNA samples

DNA samples from mock communities encoding the genes of interest were generated by mixing genomic DNA from different organisms in variable proportions, in order to simulate variation in overall GC mol% content. The pool of DNA in the mock communities included DNA from: *Nitrosospira multiformis*, *Nitrososphaera viennensis*, *Nitrospira defluvii*, Ca. *Kuenenia stuttgartiensis* PCR fragment of the full-lenght *hzoA* gene, *Pseudomonas aeruginosa*, *Escherichia coli*, *Shigella sonnei*, *Cupriavidus metallidurans*, *Cupriavidus necator*, *Dyadobacter fermentans*, *Pseudomonas stutzeri*, *Rhodobacter sphaeroides*, *Salinibacter ruber*, *Sulfurimonas denitrificans*, *Methylosinus trichosporium* Ob3b, *Methylocella tundrae*, *Methylomicrobium buryatense*, *Methanoregula boonei*, and *Methanolacinia petrolearia*. Strain information, including genome or fragment size and the number of target genes per organism are shown in Supplementary Table S1. DNA from cultured organisms was extracted from 1% sodium dodecyl sulfate (SDS)-treated cell pellets in Cetyltriammonium Bromide (CTAB) buffer, followed by phenol:chloroform:isoamyl alcohol extraction and ethanol precipitation, as described before [58]. The relative abundances of the microorganisms in the mock communities were multiplied with the median genome GC mol% contents to generate a weighted GC mol% content of the pool of samples with the following values: 47, 50, 53, 57, 60, and 63 (Supplementary Table S2). Pooling ratios of microorganisms’ DNA were calculated based on the expected number of functional genes in the extracted DNA, the DNA concentration (determined with a Qubit HS dsDNA kit (Thermo)) and genome size. Relative gene abundances in mock communities were calculated and compared to the sequences generated using targeted metagenomics. The relative abundance of each gene was calculated against the sum of abundance values of all 14 genes in the targeted metagenomes of the mock community samples (relative abundance of a specific gene = 100× (gene reads/sum of reads of 14 genes)). The captured reads for mock community targeted metagenomics are shown in Supplementary Table S4.

### Extraction of DNA and determination of chemical parameters from environmental samples

Samples from two different environments, an agricultural soil in Hungary (*n* = 3), and a wetland in Bellefontaine, France (*n* = 3) (Supplementary Table S3), were collected in order to assess the effectiveness of the probe set in enriching functional genes in different sample contexts. Extraction of DNA from the environmental samples was performed as previously described by [63]. Briefly, samples were homogenized by bead-beating 0.5 g of soil at a speed of 5.5 m/s, for 30 s with phenol:chloroform:isoamyl alcohol extraction in CTAB buffer, followed by ethanol precipitation. The quality of DNA extracts was assessed using a NanoDrop ND-1000 (Thermo), and DNA concentration was measured with a Qubit HS dsDNA kit (Thermo). The analysis of soil chemical parameters for soil C/N ratio, pH, Fe II/III-, ammonium- and nitrate content were determined as described earlier [63, 64].

### Targeted metagenomic library preparation, target enrichment of libraries and sequencing with Illumina Miseq

To prepare DNA for targeted metagenomics with probe capture, DNA was first fragmented and indexed as follows. For each sample, sequencing indexes and sequencing adapters were provided as commercial service by the Center for Genomic Research laboratories, at the University of Liverpool, Liverpool, UK. Libraries were produced with KAPA HyperPlus Library Preparation (Roche) kit to produce insert sizes of 630 bp according to the manufacturer’s instructions. The protocol is shown in Fig. 1 and described in Supplementary material and methods. Sequencing for probe hybridized and washed DNA was performed with Illumina MiSeq PE300 chemistry in the Center for Genomic Research, University of Liverpool, Liverpool, UK, resulting in 198 700–311 500 reads per sample for the environmental samples and up to 2 600 000 reads for the mock communities.

### Targeted metagenomics read processing, mapping, and evaluation of functional annotation of reads

All six possible reading frames of the nucleotide sequence reads generated by the targeted metagenomic sequencing of the mock community samples were translated using transeq from the EMBOSS package (v6.6.0.0) [65]. These were mapped to the genomes using DIAMOND blastp (v2.0.6.144) [66], with a minimum percentage identity of 60%. This threshold was selected as it resulted in the largest percentage of mapped reads while being stringent enough to prevent spurious mappings. No minimum coverage was used to account for reads that did not fully overlap with the proteins from the mock community dataset. For each direction, up to four matches were retained, to account for cases where a single read spanned neighboring proteins. Multiple matches in the same direction could overlap by up to 15 amino acids to account for possible protein fusions or sequencing errors. The blastx searches against the *refseq* database [67] were performed in order to remove duplicates or reannotate falsely annotated entries due to the presence of homologous gene families in our dataset (*amoA* vs. *pmoA*, *nxrB* vs. *narH* nitrate reductase or *napA*+*narG* vs. *nrfA*). The blastx search was done to evaluate whether the read was correct or not, and the run was made with the default parameters of blast. Each of the (up to four) matches were used to assign Kyoto Encyclopedia of Genes and Genomes (KEGG) Orthology (KO) to the reads, based on the predicted KOs of the proteins to which they mapped. If, in a given direction, a read was assigned the same KO multiple times, this was collapsed into a single hit, retaining the hit with the best E-value. Protein predictions for each read were performed using nhmmer from the HMMER suite (v.3.3) [68] using the in-house generated HMMs for target genes as well as KOFAM as described above in order to true and false detection of each gene. The script is available in Zenodo [62] /probe-capture/tree/main/3-other_scripts/Angus_scripts -folder, explained briefly in Supplementary Materials and Methods (Supplementary Fig. S5).

### Shotgun metagenomics library preparation, sequencing with Illumina HiSeq and analysis

Shotgun metagenomic sequencing was performed on environmental DNA (*n* = 3) from agricultural soil and wetland soil (Supplementary Table S3). Illumina TruSeq library preparation with an insert size of 250 bp was carried out with 1 μg of DNA and sequencing was performed in Illumina HiSeq PE150 lane as a commercial service in Vienna Biocenter Core Facility (Austria) and Microsynth AG, Switzerland. In this approach, the previously generated HMM profiles were used to identify the target gene pools (*E* < 0.001). The predicted functions of each identified sequence were confirmed by tblastx against the *refseq* database, using DIAMOND [66]. Annotation and identity information was further processed with awk in unix and in R version 3.5.3 [69] to produce lists of community structures for each target gene. The relative abundance of each gene was normalized against the sum of total reads, Supplementary Table S4: Relative abundance of gene × = (Abundance of gene ×/total reads sequenced) × 100.

### *amoA* gene amplicon sequencing

Archaeal *amoA* gene amplicons from environmental DNA (*n* = 3) from the agricultural soil were generated as described earlier by [63], and sequencing was performed with Illumina MiSeq PE250, in LGC, Münich, Germany. In brief, *amoA* gene amplicons were generated using 0.6 ng DNA template with GoTaq DNA polymerase (Promega) and 0.5 μM primers (CamoA_19F [5′-ATGGTCTGGYTWAGACG-3′] and TamoA_632R-4 [5′-GCKGCCATCCATCKRTANGTCCA-3′]) with a 5′-prime Illumina sequencing adaptor [5′-CTCTTTCCCTACACGACGCTCTTCCGATCT-3′] for both end of above primers for making the indexing possible in the sequencing service laboratory. Cycling conditions were the following: 1 min initial denaturation at 95°C, 30 s denaturation at 94°C, 30 s annealing at 60°C and 45 s extension at 72°C, with 35 cycles. PCR reactions were performed in triplicate for each sample (*n* = 3), and purified with the High Pure PCR product purification kit (Roche). Sequencing indexes were added by LGC, where libraries were equally pooled and sequenced with Illumina MiSeq PE250.

### Processing of *amoA* reads

Processing of Archaeal *amoA* (Thaumarchaeal-*amoA*, *TamoA*) amplicon, targeted- and shotgun metagenomics reads were processed by a custom-based Python script available at Zenodo [62] for amplicons /probe-capture/tree/main/1-scripts/1-amplicon_seq_script_v1.sh -folder, for probe-capture: tree/main/1-scripts/2-probe_capture_script_v1.sh -folder, and shotgun metagenomics: tree/main/1-scripts/3-metagenomics_script_v1.sh -folder, and explained briefly in Supplementary Materials and Methods.

### Phylogenetic placement analysis of *nosZ* reads from shotgun and targeted metagenomics libraries

A reference alignment and phylogeny for *nosZ* was generated from full length *nosZ* amino acid sequences. Script available for *nosZ* phylogenetic placements of metagenomic reads at Zenodo [62] /probe-capture/tree/main/3-other_scripts/Chris_Jones_scripts -folder, and explained briefly in Supplementary Materials and Methods.

### Data and script availability

The shotgun metagenomic, targeted metagenomic, and *amoA* amplicon sequencing data have been deposited in NCBI SRA under the Bioproject numbers PRJNA898102 and PRJNA488558. All scripts are available at Zenodo [62]. The repository contains the target gene database, probe-capture/2-selected_outputs/all_genes_compiled_ncbi_nt_envnt_fungene_split_file_0.fasta.gz -file (four files ..file_0-3.fasta.gz), and the final probe sequences probe-capture/2-selected_outputs/Final_N_probes.list1.fasta -file.

## Results

### Benchmarking gene detection and quantification using targeted metagenomics in mock communities

**Figure 2:**
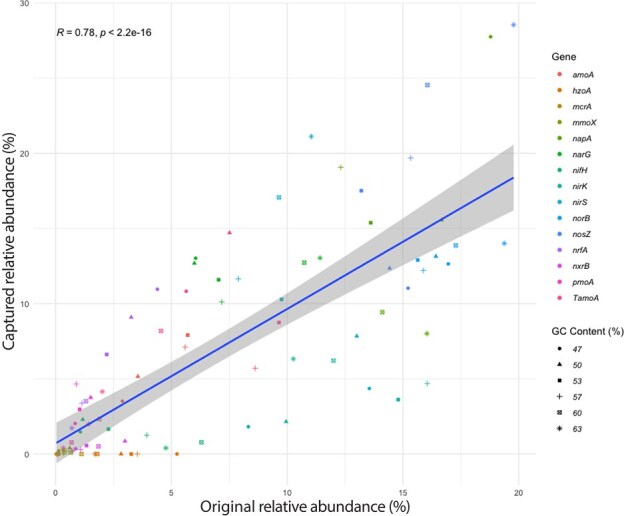
Comparison between the original composition (DNA of strains applied into the mock sample) of the mock community and the relative abundance for each functional gene detected using probe captured metagenomics for all GC mol% combinations combined. The relative abundance in the mock community was calculated based on the amount of each functional gene and organism as well as genome size. Color of the symbols depict gene and shape depicts the category of GC content (mol%).

The targeted metagenomic approach was evaluated using mock microbial communities comprising predefined mixtures of genomic DNA from 14 bacterial and three archaeal strains, together containing all 14 functional genes targeted by the probes and mixed in different proportions to produce six different median G + C contents ranging from 47 mol% to 62 mol% (Supplementary Table S1). The overall relative abundances of target genes in the captured metagenomes across all GC mol% categories were correlated with their abundance in the original mock communities (*r* = 0.78, *P* < .0001) (Fig. 2). Comparisons between the relative abundance of individual genes in the mock communities as compared to captured metagenomes revealed highly similar values (*t* = 1.8518e−10, df = 174.83, *P* > .05), with the exception of the *nirK* and the *hzoA* genes (Supplementary Fig. S1A and B). Pearson correlation coefficients across all GC% categories varied between 0.76 and 0.93, although correlations were stronger within higher GC mol% categories (57%–63%; *R* = 0.80–0.93) than within lower GC mol% categories (47%–53%; *R* = 0.76–0.79) (Supplementary Fig. S1). The probe capture targeted metagenomics produced 61–72% of target gene sequences from the total sequenced library of the mock communities (Supplementary Table S4).

### Precision of gene identification in mock communities

Precision for the in-house HMMs ranged from 74.1% (*nifH*) to 100% (*mcrA*) (Supplementary Fig. S4). The average precision of the models was 93.4%, with a median of 99.8% (excluding *amoA* and *pmoA*). The recall ranged from 42.8% (*nxrB*) to 100% (*mcrA*). Because KEGG only provides a single model covering the homologous genes *pmoA* and *amoA*, it was not possible to accurately calculate the precision and recall for these genes individually. A precision estimate could still be determined for the two genes, where a “true positive” is defined as a hit for a read identified by the in-house models as *amoA* or *pmoA* mapped to a coding sequence identified by the HMM for (*a*/*p*)*moA* genes provided by KEGG (K10944). In this case, the precision values for the *amoA* and *pmoA* genes were estimated to be 96.9% and 100%, respectively. It is important to note that these estimates do not exclude the possibility that the in-house models misclassified reads belonging to these homologous protein families. Precision and recall values were similar using KOFAMs instead of in-house HMMs. However, the results for *amoA* and *pmoA* genes were combined due to a single model for the associated KO for both genes, as mentioned above. The precision of KOs was lower for *napA* than for other genes when compared with custom DNA HMMs. This could be accounted for by the presence of formate dehydrogenase, a known homolog of *napA* [70].

### Higher number of reads for all target functional genes but similar relative abundance obtained with targeted compared to shotgun metagenomics from complex environmental samples

**Figure 3:**
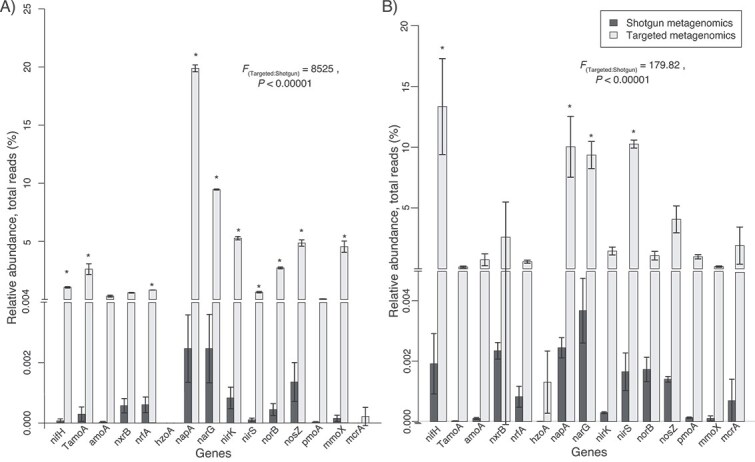
The relative abundance of functional genes (calculated against total sequencing reads) involved in inorganic nitrogen and methane cycling obtained by targeted metagenomics and shotgun metagenomics from (A) agricultural soil and (B) wetland soil. Relative abundance was retrieved with HMMER [71] searches of each functional gene, analytical script available in Zenodo [62] and explained briefly in Supplementary methods. Statistically significant differences between shotgun and targeted relative abundances according to pairwise comparisons with ANOVA are shown with asterisk (*P* < .05, *n* = 3). The comparison of shotgun vs. targeted metagenomic measured with ANOVA is shown as *F* values and significance in the inset for both samples.

We investigated the efficiency of the targeted metagenomics approach in natural complex ecosystems by directly comparing this approach with shotgun untargeted metagenomics generated with a nearly 100 times higher amount of sequencing (~66 Mb and ~6.4 Gb per sample, respectively) from wetland soils in Bellefontaine, France, and from an agricultural field in Hungary (Supplementary Table S4). These soils represent distinct ecosystems with average physicochemical conditions, such as water content and oxygen concentration, and N availability that favors different distributions of functional genes [12]. Despite the large difference in sequencing depth, targeted metagenomics generated a much larger set of target functional genes (Fig. 3). In both the agricultural and wetland site, the targeted metagenomics approach detected 14 out of 15 gene-clusters and the shotgun approach 13 out of 15 genes (*mcrA* was not detected in the agricultural site and *hzoA* not in the wetland site). Moreover, up to 60 times as many identified gene sequences were detected using the probe capture compared to shotgun metagenomics (Fig. 3, Supplementary Table S4). The relative abundances of functional genes in captured metagenomes correlated significantly with that in shotgun metagenomes from both the agricultural field (*R*_Pearson_ = 0.96, d.f. = 14, *P* < .00001) and the wetland (*R*_Pearson_ = 0.70, d.f. = 14, *P* < .01). The relative abundances of target genes were different between the two approaches (*P* > .00001) (Fig. 3). Moreover, increased relative abundance was detected with targeted compared to shotgun metagenomics in the agricultural site for *nifH*, *TamoA*, *nrfA*, *napA*, *nirK*, *nirS*, *norB*, *nosZ,* and *mmoX* (Fig. 3A) genes and the wetland site for *nifH*, *napA, narG,* and *nirS* (Fig. 3B) genes. The targeted approach generated a much higher number of reads for all target genes in both sites than shotgun metagenomics (Target reads in Supplementary Table S4).

The different relative abundances of functional genes in the two ecosystems reflected known differences in their environmental conditions: (i) N_2_ fixation genes were expectedly higher in the unfertilized wetland, (ii) Genes involved in ammonia oxidation and nitric and N_2_O reduction were higher in the agricultural soil, which is likely to be more oxic and also subject to N fertilization which increase inorganic N cycling, (iii) Genes involved in methanogenesis were more abundant in the wetland, where anoxic conditions and CH_4_ production typically occur.

### Comparing diversity recovered for specific target genes from amplicon, targeted and shotgun metagenomics from complex environmental samples

**Figure 4:**
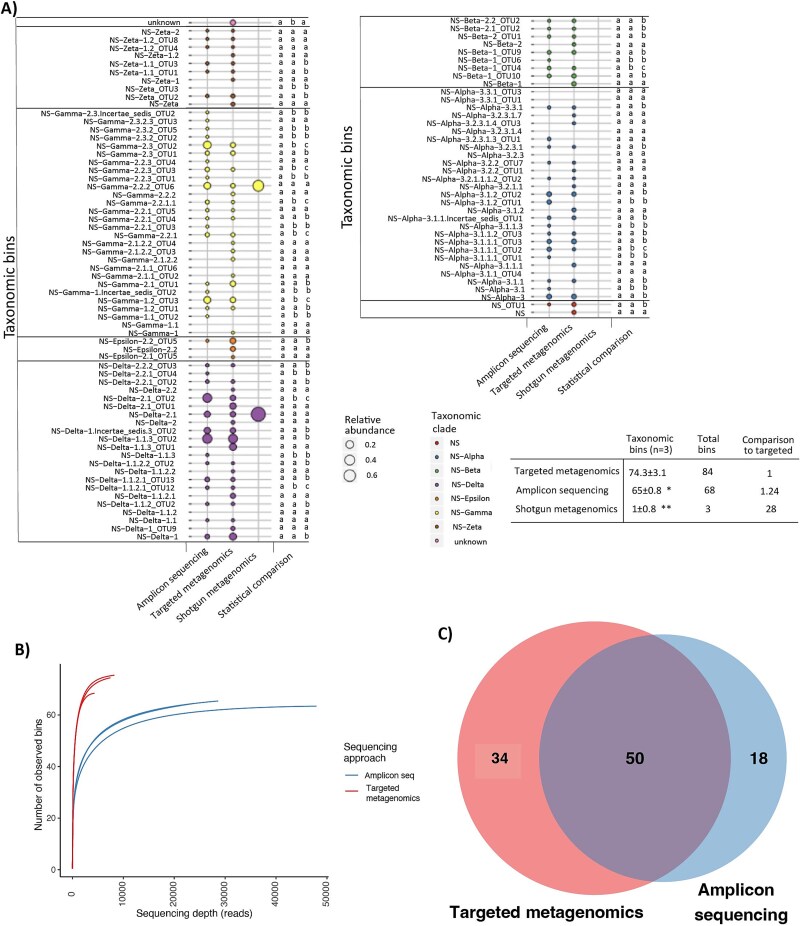
(A) The relative abundance of archaeal *amoA* taxonomic bins produced from amplicon sequencing with *amoA* amplicon sequencing (*amoA* gene PCR amplicons sequenced with Illumina MiSeq, about 22 000–47 000 *amoA* reads per sample) (*n* = 3), metagenomics reads of targeted metagenomics (genomic DNA sequenced with Illumina MiSeq, after target enrichment, about 4500–8500 *amoA* reads out of 220 000–310 000 targeted reads per sample)(*n* = 3), or shotgun metagenomics (genomic DNA sequenced with Illumina HiSeq, about 22–57 million reads per sample, of which only two were archaeal *amoA*)(*n* = 3). In all cases, the analysis pipeline consisted of classifying the generated reads with USEARCH [74] using the *amoA* reference database [26], while taxonomy was analyzed with the QIIME1 [75] analytical pipeline. The script is available in Zenodo [62] and explained briefly in Supplementary methods. The sample having the largest diversity is shown (*n* = 3). The bubble symbol size is proportional to the relative abundance of each taxonomic bin produced with the sequencing approach indicated in each column.. The color indicates the clade affiliation of each taxonomic bin within Nitrososphaerales. Statistically significant differences in the abundance of each taxonomic bin between sequencing approaches based on pairwise-Wilcox comparison are indicated with different letters (*P* < .05, *n* = 3), and the significant difference of comparison for taxonomic bins is shown with an asterisk, ^*^P < .05, ^**^P < .01. (B) Rarefaction analysis of archaeal *amoA* obtained by amplicon sequencing and captured metagenomics. (C) Venn diagram of archaeal *amoA* sequences obtained by amplicon sequencing and targeted metagenomics.

In order to compare the diversity of specific functional genes detected by targeted metagenomics, shotgun metagenomics and PCR amplicon sequencing, we focused on the archaeal *amoA* gene as a test case, as it is the second most sequenced gene in NCBI indicative of its ecological importance, and for which there is a comprehensive reference database for classification available [26]. Moreover, archaeal ammonia oxidizers typically outnumber their bacterial counterparts in most environments [71], and even though they can be low abundant, their diversity is striking and underlines their distinct metabolic capabilities [26, 72]. Additionally, we compared the detection of *nosZ* genes between the targeted and shotgun metagenomics approaches in the agricultural soil, also based on an existing reference database [73]. The following datasets were compared: (i) targeted and shotgun metagenomes, and PCR amplicons of the archaeal *amoA* gene in the agricultural soil (Fig. 4), (ii) *nosZ* gene sequences identified by targeted or shotgun metagenomics in the agricultural soil (Supplementary Fig. S3).

Comparison of results from all three methods in agricultural soil samples showed that targeted metagenomics detected a substantially higher diversity of archaeal *amoA* genes than PCR amplicon sequencing, while, as expected, shotgun metagenomics detected a lower diversity than either of the other two methods (Fig. 4A). Specifically, targeted metagenomics detected 71–83 distinct taxonomic bins of *amoA* gene, whereas PCR amplicon sequencing and shotgun metagenomics detected 65–68 taxonomic bins and only three bins, respectively. Rarefaction analysis also indicated that targeted metagenomics retrieved a higher alpha diversity of archaeal *amoA* genes than PCR amplicon sequencing (Fig. 4B and C) and it also reaches a plateau much faster and with a lower number of sequences than amplicon sequencing (Fig. 4B).

Similarly, targeted metagenomes yielded a higher number of taxonomic bins for *nosZ* genes from the agricultural soil (~6800 bins) than shotgun metagenomics (~1300 bins) at a 90% identity cutoff, showing that the first approach detected a greater gene diversity with a much lower sequencing depth (Supplementary Fig. S3). Gene sequence reads from both methods mapped to similar lineages in a comprehensive reference phylogeny of *nosZ* genes [76]. However, a greater proportion of reads from the targeted metagenomics approach were assigned to deeper nodes of the phylogeny, particularly within the less studied clade II, suggesting that this method also captured more novel *nosZ* variants not represented in the reference phylogeny.

Targeted metagenomics (sequencing depth 117–160 Mb/sample) yielded 29.1 ± 3.1 *TamoA* reads per 100 000 total reads, while shotgun metagenomics (7–17 Gb/sample) yielded only 0.0036 ± 0.0039. The shotgun metagenomics produced only three quality-controlled *amoA* taxonomic bins were detected (Fig. 4A). Conversely, targeted metagenomics generated five times less *TamoA* reads (Fig. 4B) than PCR amplicon sequencing, with a sequencing depth of 15–31 Mb per sample. Nevertheless, the targeted metagenomics approach detected a much higher gene sequence diversity for both *TamoA* (Fig. 4A and B) and *nosZ* (Supplementary Fig. S3) than the two other methods in the agricultural soil samples. For *TamoA*, the targeted approach detected 28 times higher alpha-diversity than shotgun metagenomics, while 5 times higher diversity of *nosZ* genes was recovered, importantly covering genes affiliated with the less characterized Clade II. In turn, the targeted approach detected 1.24 times (24% more taxonomic bins) higher diversity of archaeal *amoA* genes than PCR amplicon sequencing in agricultural soils, respectively.

## Discussion

In this study, we report the development and evaluation of a new probe library for targeted metagenomics of 14 key genes involved in the microbial cycling of inorganic N and CH_4_. This included the compilation of databases containing all variants of the target genes from public databases, the development of an extensive set of probes targeting 14 well-established marker genes, and a bioinformatics pipeline to process and annotate the sequence data. This approach has been successfully used earlier to identify genes involved in microbial C metabolism in soil [53, 77].

In validation experiments with mock communities comprising 18 distinct organisms encoding multiple target genes, we showed that this method successfully reproduced the expected relative abundances of nearly all target genes, with the exception of the *nirK* and *hzoA* genes. Although 10.3% of the probes targeted *nirK* genes of nitrifiers*,* these were detected at a lower relative abundance than that present in the original mock community. However, the relative abundance of *nirK* genes matched the frequency of *nirK* genes only in heterotrophic organisms in the mock community, indicating a bias in the detection of distinct *nirK* gene variants. The sequencing technology used can influence the sequences generated due to known GC biases. In this case, the relative abundance of *nirK* genes may have been biased by the false low coverage of genomic regions with non-optimal GC mol% content (50%–60%) known from MiSeq Illumina sequencing [78]. Consistent with this observation, there was some variation in probe specificity among mock communities with different genomic GC mol% content, with high GC mol% communities having a generally higher target gene detection efficiency. For example, the mean GC% content of nitrifier *nirK* genes is lower than that of heterotrophic *nirK* genes (53% and >60%, respectively), and thus it is plausible that the later are sequenced more efficiently. This effect was also noticeable among *nosZ* genes, as the *nosZ* gene of *D. fermentas*, with GC content of 52.9 mol% (Supplementary Table S1, Supplementary Fig. S1), was detected more efficiently than that of *S. denitrificans*, which has a 32.7 mol% GC content.

Targeted metagenomics also closely reproduced the relative abundances of target genes obtained with shotgun metagenomics in two distinct natural microbial communities from soils. The relative gene abundances were strongly positively correlated between the two methods, indicating that the probe capture step did not introduce a significant quantitative bias. Importantly, this relationship held also true for *nirK* genes, confirming that the lower detection efficiency of nitrifier *nirK* genes was due to a variation in GC content. This observation emphasizes that particular attention should be given to the design of probes for low GC genes, such as by targeting gene regions that are not highly conserved among all gene variants, where higher GC probes are more likely to outcompete lower GC probes, as in the case of *nirK*. Nonetheless, as observed in the mock communities, the relative gene abundances quantified in environmental samples were strongly positively correlated between the two approaches to all GC mol% categories (Supplementary Fig. S2). This further supports that the observed variation in detection efficiencies among gene variants with targeted metagenomics has a negligible effect on community profiles.

The thaumarchaeal *amoA* gene (*TamoA*) and the *nosZ* gene were used as case studies to investigate the differences in the detected diversity of single functional guilds that are known to be highly diverse [26, 73]. To determine the community composition of the *TamoA* gene for the different sequencing approaches, the reads are mapped to a reference library, in turn forming the taxonomic bins. Consequently, the same gene from the same organism can be detected multiple times in targeted metagenomics due to multiple probe hybridization. However, when calculation of relative abundance is performed, it balances the detected community because an equal number of probes is used per sequence cluster and therefore the relative abundance of targeted method can be compared to other sequencing tools. At first glance, PCR amplicon sequencing seems to have resulted in 18 more taxonomic bins than targeted metagenomics. However, these sequences were generated by a 35-cycle PCR, known to produce some chimeric sequences. Examples of such errors have been shown even for shorter amplification cycles [79]. Problems due to chimeric sequences in public databases from amplicon studies are known and were encountered during the compilation of the thaumarchaeal *amoA* reference database [26]. Although we used standard pipelines for the detection of chimeric sequences, it is possible that we might have missed some, which may be represented in the 18 bins produced with amplicon sequencing, leading to an inflated number of sequences post-processing. The absence of long amplification steps in targeted metagenomics is expected to preclude the formation of chimeric sequences, which represents a significant advantage over typical PCR-based approaches.

It should be noted that the efficiency of the targeted metagenomic approach largely depends on the coverage and quality of the database used to generate the probes. We used hmmer profiles to collect all possible genes from the NCBI nucleotide and WGS databases, as well as Fungene and other published databases were used for the target gene library. Only one hmmer profile per gene was used for detection, however, clade specific hmmer profiles could also be used to have a broader outcome for neighboring clades. We used fairly relaxed conditions when including thresholds for screening the NCBI databases, because we wanted to have closely related organisms and genes in the probe pool. For example, this was the case for the *mmoX* gene family, which also includes butane, propane, and toluene monooxygenases. We included these closely related *mmoX* genes in the probes, therefore these closely related *mmoX* genes were found from coniferous trees [76]. However, the database is not constrained to full-length gene sequences from a limited diversity of complete genomes and long metagenomic scaffolds available and the probe/target diversity can be greatly extended through the inclusion of gene fragments generated by PCR or from short metagenomic contigs. Despite its high target precision, this method may nevertheless capture non-target sequences, especially for orthologous gene families who have evolved different functions, as is the case for *napA* genes and formate dehydrogenases. Such cases emphasize the importance of thorough data annotation and filtering procedures. With the target gene database used to generate probes in this study, we managed to detect a higher diversity for the case study of *TamoA* genes than the shotgun sequencing approach, showcasing the advantage of “casting a wider net” with targeted metagenomics. Similar results were obtained in other targeted metagenomics applications, such as for detection of diverse resistome-virulome elements [54] or for improved taxonomic microbial community characterization via 16S rRNA enrichment [55].

Despite the technological advances and decline in cost of high-throughput sequencing, shotgun metagenomics remain impractical and prohibitively expensive to capture the diversity of specific, low abundant functional groups in complex environments, such as soils and sediments, especially in longitudinal studies. The current cost of generating the probes for targeted metagenomics is about 20–50 € per sample depending on the probe manufacturer. If the goal is to have a focused, comprehensive view of the diversity of functional guilds involved in inorganic nitrogen or CH_4_ cycling in a certain ecosystem, then targeted metagenomics can circumvent the high cost and overabundance of data generated by shotgun metagenomics, as well as provide more quantitative data and more information on the diversity of the genes of interest. Thus, this approach has not only the potential to capture rare and novel gene diversity in complex environments, but also to identify cryptic microorganisms in low-biomass samples or involved in suggested CH_4_ metabolisms, such as in the tree phyllosphere [76] and nitrogen cycling in coral holobiont [80]. Moreover, targeted metagenomics can also overcome issues associated with running and comparing multiple independent PCR assays when investigating several distinct targets. In that sense, this approach effectively represents a PCR-independent, multiplex approach to characterize simultaneously and in-depth the distributions of a broad range of functional genes, providing a holistic view of the status of the nitrogen and CH_4_ cycles in the studied ecosystems. This is especially advantageous when combined with functional studies, such as the determination of N-transformation rates and in situ fluxes, as showcased by a study of N_2_O emissions in thawing Yedoma permafrost sites over time [81]. In this study, the application of targeted metagenomics with the N-cycling probe dataset presented here revealed that changes in the N-cycling microbial community composition were responsible for an increase in N_2_O emissions in revegetated Yedoma soils, which had undergone thawing a half decade prior.

Conclusively, the targeted metagenomics approach developed here provides an efficient and cost-effective strategy for studying microbial functional guilds that typically represent small fractions of natural microbiomes, and whose diversity is generally underestimated and highly underrepresented in metagenomic datasets. This approach also circumvents the limitations and biases associated with PCR-based methods and has higher potential to capture rare or novel functional gene diversity.

## Supplementary Material

Supplementary_Fig_S1

Supplementary_Fig_S2

Supplementary_Fig_S3

Supplementary_Fig_S4

Supplementary_Fig_S5

Supplementary_Table_S1

Supplementary_Table_S2

Supplementary_Table_S3

Supplementary_Table_S4

Siljanen_etal_2025_ycaf183_Supplementary_Materials

## References

[1] Falkowski P, Scholes RJ, Boyle E. et al. The global carbon cycle: a test of our knowledge of earth as a system. *Science* 2000;290:291–6. 10.1126/science.290.5490.291 11030643

[2] Gruber N, Galloway J. An earth-system perspective of the global nitrogen cycle. *Nature* 2008;451:293–6. 10.1038/nature06592 18202647

[3] Archer D . The Global Carbon Cycle. Princeton: Princeton University Press, 2010.

[4] Zaehle S . Terrestrial nitrogen–carbon cycle interactions at the global scale. *Phil Trans R Soc* 2013;368:B3682013012520130125. 10.1098/rstb.2013.0125 PMC3682745 23713123

[5] Steffen W, Richardson K, Rockström J. et al. Planetary boundaries: guiding human development on a changing planet. *Science* 2015;347:1259855. 10.1126/science.1259855 25592418

[6] Galloway JN, Dentener FJ, Capone DG. et al. Nitrogen cycles: past, present, and future. *Biogeochemistry* 2004;70:153–226. 10.1007/s10533-004-0370-0

[7] Offre P, Spang A, Schleper C. Archaea in biogeochemical cycles. *Ann Rev Microbiol* 2013;67:437–57. 10.1146/annurev-micro-092412-155614 23808334

[8] Stein LY, Klotz MG. The nitrogen cycle. *Curr Biol* 2016;26:R94–8. 10.1016/j.cub.2015.12.021 26859274

[9] Kuypers M, Marchant H, Kartal B. The microbial nitrogen-cycling network. *Nat Rev Microbiol* 2018;16:263–76. 10.1038/nrmicro.2018.9 29398704

[10] Barnard R, Leadley PW, Hungate BA. Global change, nitrification, and denitrification: a review. *Glob Biogeochem Cycles* 2005;19:GB1007. 10.1029/2004GB002282

[11] Thomson RAJ, Giannopoulos G, Pretty J. et al. Biological sources and sinks of nitrous oxide and strategies to mitigate emissions. *Philos Trans R Soc B* 2012;367:1157–68. 10.1098/rstb.2011.0415 PMC3306631 22451101

[12] Jones C, Spor A, Brennan F. et al. Recently identified microbial guild mediates soil N_2_O sink capacity. *Nature Clim Change* 2014;4:801–5. 10.1038/nclimate2301

[13] Prosser JI, Hink L, Gubry-Rangin C. et al. Nitrous oxide production by ammonia oxidizers: physiological diversity, niche differentiation and potential mitigation strategies. *Glob Change Biol* 2020;26:103–18. 10.1111/gcb.14877 31638306

[14] Bodelier PLE, Laanbroek HJ. Nitrogen as a regulatory factor of methane oxidation in soils and sediments. *FEMS Microbiol Ecol* 2004;47:265–77. 10.1016/S0168-6496(03)00304-0 19712315

[15] Conrad R . The global methane cycle: recent advances in understanding the microbial processes involved. *Environ Microbiol Rep* 2009;1:285–92. 10.1111/j.1758-2229.2009.00038.x 23765881

[16] Murrell JC, Jetten MSM. The microbial methane cycle. *Environ Microb Rep* 2009;1:279–84. 10.1111/j.1758-2229.2009.00089.x 23765880

[17] Intergovernmental Panel on Climate Change (IPCC) . Climate Change 2013—The Physical Science Basis: Working Group I Contribution to the Fifth Assessment Report of the Intergovernmental Panel on Climate Change. Cambridge: Cambridge University Press, 2014. 10.1017/CBO9781107415324

[18] Friedlingstein P, O’Sullivan M, Jones MW. et al. Global carbon budget. *Earth Syst Sci Data* 2020;12:3269–340. 10.5194/essd-12-3269-2020

[19] Lan X, Thoning KW, Dlugokencky EJ. Trends in Globally-Averaged CH4, N2O, and SF6 Determined from NOAA Global Monitoring Laboratory Measurements. Version 2023-09. 10.15138/P8XG-AA10

[20] Håvelsrud OE, Haverkamp TH, Kristensen T. et al. A metagenomic study of methanotrophic microorganisms in coal oil point seep sediments. *BMC Microbiol* 2011;11:221. 10.1186/1471-2180-11-221 21970369 PMC3197505

[21] Palmer K, Biasi C, Horn MA. Contrasting denitrifier communities relate to contrasting N_2_O emissions patterns from acidic peat soils in arctic tundra. *ISME J* 2021;6:1058–77. 10.1038/ismej.2011.172 PMC3329112 22134649

[22] Aanderud ZT, Jones SE, Fierer N. et al. Resuscitation of the rare biosphere contributes to pulses of ecosystem activity. *Front Microbiol* 2015;6:1–11. 10.3389/fmicb.2015.00024 PMC4311709 25688238

[23] Nelson MB, Martiny AC, Martiny JBH. Global biogeography of microbial nitrogen-cycling traits in soil. *PNAS* 2016;113:8033–40. 10.1073/pnas.1601070113 27432978 PMC4961168

[24] Ouyang Y, Norton JM. Nitrite oxidizer activity and community are more responsive than their abundance to ammonium-based fertilizer in an agricultural soil. *Front Microbiol* 2020;11:1–10. 10.3389/fmicb.2020.01736 PMC7417772 32849372

[25] Alteio LV, Séneca J, Canarini A. et al. A critical perspective on interpreting amplicon sequencing data in soil ecological research. *Soil Biol Biochem* 2021;160:108357. 10.1016/j.soilbio.2021.108357

[26] Alves RJE, Minh BQ, Urich T. et al. Unifying the global phylogeny and environmental distribution of ammonia-oxidizing archaea based on *amoA* genes. *Nat Commun* 2018;9:1517. 10.1038/s41467-018-03861-1 29666365 PMC5904100

[27] Borrel G, Adam PS, McKay LJ. et al. Wide diversity of methane and short-chain alkane metabolisms in uncultured archaea. *Nat Microbiol* 2019;4:603–13. 10.1038/s41564-019-0363-3 30833729 PMC6453112

[28] Li J, Liu T, McIlroy SJ. et al. Phylogenetic and metabolic diversity of microbial communities performing anaerobic ammonium and methane oxidations under different nitrogen loadings. *ISME Commun* 2023;3:39. 10.1038/s43705-023-00246-4 37185621 PMC10130057

[29] Saghaï A, Pold G, Jones CM. et al. Phyloecology of nitrate ammonifiers and their importance relative to denitrifiers in global terrestrial biomes. *Nat Commun* 2023;14:8249. 10.1038/s41467-023-44022-3 38086813 PMC10716430

[30] Pan Y, Bodrossy L, Frenzel P. et al. Impacts of inter- and intralaboratory variations on the reproducibility of microbial community analyses. *Appl Environ Microbiol* 2010;76:7451–8. 10.1128/AEM.01595-10 20870788 PMC2976186

[31] Nnadozie CF, Lin J, Govinden R. Selective isolation of bacteria for metagenomic analysis: impact of membrane characteristics on bacterial filterability. *Biotechnol Prog* 2015;31:853–66. 10.1002/btpr.2109 26018114

[32] Sinha R, Abu-Ali G, Vogtmann E. et al. Assessment of variation in microbial community amplicon sequencing by the microbiome quality control (MBQC) project consortium. *Nature Biot* 2017;35:1077–86. 10.1038/nbt.3981 PMC5839636 28967885

[33] Sui HY, Weil AA, Nuwagira E. et al. Impact of DNA extraction method on variation in human and built environment microbial community and functional profiles assessed by shotgun metagenomics sequencing. *Front Microb* 2020;11:1–16. 10.3389/fmicb.2020.00953 PMC7262970 32528434

[34] Orellana LH, Hatt JK, Iyer R. et al. Comparing DNA, RNA and protein levels for measuring microbial dynamics in soil microcosms amended with nitrogen fertilizer. *Sci Rep* 2019;9:17630. 10.1038/s41598-019-53679-0 31772206 PMC6879594

[35] Zhao J, Huang L, Chakrabarti S. et al. Nitrogen and phosphorous acquisition strategies drive coexistence patterns among archaeal lineages in soil. *ISME J.* 2023;17:1839–50. 10.1038/s41396-023-01493-y 37596409 PMC10579303

[36] Knief C . Diversity and habitat preferences of cultivated and uncultivated aerobic methanotrophic bacteria evaluated based on *pmoA* as molecular marker. *Front Microbiol* 2015;6:1346. 10.3389/fmicb.2015.01346 26696968 PMC4678205

[37] Bräuer SL, Basiliko N, Siljanen HMP. et al. Methanogenic archaea in peatlands. *FEMS Microbiol Lett* 2021;367:1–17. 10.1093/femsle/fnaa172 33068423

[38] Romdhane S, Spor A, Aubert J. et al. Unraveling negative biotic interactions determining soil microbial community assembly and functioning. *ISME J* 2021;16:296–306. 10.1038/s41396-021-01076-9 34321619 PMC8692615

[39] Clark DR, McKew BA, Binley A. et al. Hydrological properties predict the composition of microbial communities cycling methane and nitrogen in rivers. *ISME Commun* 2022;2:5. 10.1038/s43705-022-00087-7 37938696 PMC9723640

[40] Hallin S, Lindgren PE. PCR detection of genes encoding nitrite reductase in denitrifying bacteria. *Appl Environ Microbiol* 1999;65:1652–7. 10.1128/AEM.65.4.1652-1657.1999 10103263 PMC91233

[41] Throbäck IN, Enwall K, Jarvis Å. et al. Reassessing PCR primers targeting nirS, nirK and nosZ genes for community surveys of denitrifying bacteria with DGGE. *FEMS Microbiol Ecol* 2004;49:401–17. 10.1016/j.femsec.2004.04.011 19712290

[42] Kolb S, Knief C, Stubner S. et al. Quantitative detection of methanotrophs in soil by novel pmoA-targeted real-time PCR assays. *Appl Environ Microbiol* 2003;69:2423–9. 10.1128/AEM.69.5.2423-2429.2003 12732507 PMC154495

[43] Siljanen HMP, Saari A, Krause S. et al. Hydrology is reflected in the functioning and community composition of methanotrophs in the littoral wetland of a boreal lake. *FEMS Microbiol Ecol* 2011;75:430–45. 10.1111/j.1574-6941.2010.01015.x 21175697

[44] Bonilla-Rosso G, Wittorf L, Jones C. et al. Design and evaluation of primers targeting genes encoding NO-forming nitrite reductases: implications for ecological inference of denitrifying communities. *Sci Rep* 2016;6:39208. 10.1038/srep39208 27966627 PMC5155301

[45] Tveit AT, Urich T, Frenzel P. et al. Metabolic and trophic interactions modulate methane production by Arctic peat microbiota in response to warming. *Proc Natl Acad Sci USA* 2015;112:E2507–16. 10.1073/pnas.1420797112 25918393 PMC4434766

[46] Futema M, Plagnol V, Whittall RA. et al. Use of targeted exome sequencing as a diagnostic tool for familial hypercholesterolaemia. *J Med Genet* 2012;49:644–9. 10.1136/jmedgenet-2012-101189 23054246 PMC3475071

[47] Skoglund P, Northoff BH, Shunkov MV. et al. Separating endogenous ancient DNA from modern day contamination in a Siberian Neandertal. *Proc Natl Acad Sci USA* 2014;111:2229–34. 10.1073/pnas.1318934111 24469802 PMC3926038

[48] Gasc C, Peyretaillade E, Peyret P. Sequence capture by hybridization to explore modern and ancient genomic diversity in model and nonmodel organisms. *Nucleic Acids Res* 2016;44:4504–18. 10.1093/nar/gkw309 27105841 PMC4889952

[49] Ichida H, Morita R, Shirakawa Y. et al. Targeted exome sequencing of unselected heavy-ion beam-irradiated populations reveals less-biased mutation characteristics in the rice genome. *Plant J* 2019;98:301–14. 10.1111/tpj.14213 30584677 PMC6850588

[50] Bewicke-Copley F, Arjun Kumar E, Palladino G. et al. Applications and analysis of targeted genomic sequencing in cancer studies. *Comput Struct Biotechnol J* 2019;17:1348–59. 10.1016/j.csbj.2019.10.004 31762958 PMC6861594

[51] Denonfoux J, Parisot N, Dugat-Bony E. et al. Gene capture coupled to high-throughput sequencing as a strategy for targeted metagenome exploration. *DNA Res* 2013;20:185–96. 10.1093/dnares/dst001 23364577 PMC3628448

[52] Kamil G, Yoon JY, Yoo S. et al. Clinical relevance of targeted exome sequencing in patients with rare syndromic short stature. *Orphanet J Rare Dis* 2021;16:297. 10.1186/s13023-021-01937-8 34217350 PMC8254301

[53] Manoharan L, Kushwaha SK, Hedlund K. et al. Captured metagenomics: large-scale targeting of genes based on ‘sequence capture’ reveals functional diversity in soils. *DNA Res* 2015;22:451–60. 10.1093/dnares/dsv026 26490729 PMC4675713

[54] Noyes NR, Weinroth ME, Parker JK. et al. Enrichment allows identification of diverse, rare elements in metagenomic resistome-virulome sequencing. *Microbiome* 2017;5:142. 10.1186/s40168-017-0361-8 29041965 PMC5645900

[55] Beaudry MS, Wang J, Kieran TJ. et al. Improved microbial community characterization of 16S rRNA via metagenome hybridization capture enrichment. *Front Microbiol* 2021;12:12. 10.3389/fmicb.2021.644662 PMC8110821 33986735

[56] Liu F, Zhang J, Mei Y. The origin of the cooperativity in the streptavidin-biotin system: a computational investigation through molecular dynamics simulations. *Sci Rep* 2016;6:27190. 10.1038/srep27190 27249234 PMC4888747

[57] Kushwaha SK, Manoharan L, Meerupati T. et al. MetCap: a bioinformatics probe design pipeline for large-scale targeted metagenomics. *BMC bioinformatics* 2015;16:65. 10.1186/s12859-015-0501-8 25880302 PMC4355349

[58] Jones CM, Graft DRH, Bru D. et al. The unaccounted yet abundant nitrous oxide-reducing microbial community: a potential nitrous oxide sink. *ISME J* 2013;7:417–26. 10.1038/ismej.2012.125 23151640 PMC3554408

[59] Graf DR, Jones CM, Hallin S. Intergenomic comparisons highlight modularity of the denitrification pathway and underpin the importance of community structure for N_2_O emissions. *PLoS One* 2014;9:e114118. 10.1371/journal.pone.0114118 25436772 PMC4250227

[60] Fish JA, Chai B, Wang Q. et al. FunGene: the Functional Gene Pipeline and Repository. *Front Microbiol* 2013;4:291. 24101916 10.3389/fmicb.2013.00291 PMC3787254

[61] Wheeler TJ, Eddy SR. nhmmer: DNA homology search with profile HMMs. *Bioinformatics* 2013;29:2487–9. 10.1093/bioinformatics/btt403 23842809 PMC3777106

[62] Bagnoud A, Siljanen H. alex-bagnoud/probe-capture: v2025-06. Zenodo, 2025. 10.5281/zenodo.15752134

[63] Siljanen HMP, Alves RJE, Ronkainen JG. et al. Archaeal nitrification is a key driver of high nitrous oxide emissions from arctic peatlands. *Soil Biol Biochem* 2019;137:107539. 10.1016/j.soilbio.2019.107539

[64] Bagnoud A, Guye-Humbert S, Schloter-Hai B. et al. Environmental factors determining distribution and activity of anammox bacteria in minerotrophic fen soils. *FEMS Microb Ecol* 2020;96:fiz191. 10.1093/femsec/fiz191 31782780

[65] Rice P, Longden I, Bleasby A. EMBOSS: the European molecular biology open software suite. *Trends Genet* 2000;16:276–7. 10.1016/s0168-9525(00)02024-2 10827456

[66] Buchfink B, Xie C, Huson DH. Fast and sensitive protein alignment using DIAMOND. *Nat Methods* 2015;12:59–60. 10.1038/nmeth.3176 25402007

[67] Altschul SF, Gish W, Miller W. et al. Basic local alignment search tool. *J Mol Biol* 1990;215:403–10. 10.1016/S0022-2836(05)80360-2 2231712

[68] Eddy SR . Accelerated profile HMM searches. *PLOS Comp Biol* 2011;7:e1002195. 10.1371/journal.pcbi.1002195 PMC3197634 22039361

[69] R Core Team . R: A Language and Environment for Statistical Computing. Vienna: R Foundation for Statistical Computing, 2018, https://www.R-project.org/.

[70] Cerqueira NMFSA, Gonzalez PJ, Fernandes PA. et al. Periplasmic nitrate reductase and formate dehydrogenase: similar molecular architectures with very different enzymatic activities. *Acc Chem Res* 2015;48:2875–84. 10.1021/acs.accounts.5b00333 26509703

[71] Leininger S, Urich T, Schloter M. et al. Archaea predominate among ammonia-oxidizing prokaryotes in soils. *Nature* 2006;442:806–9. 10.1038/nature04983 16915287

[72] Huang L, Chakrabarti S, Cooper J. et al. Ammonia-oxidizing archaea are integral to nitrogen cycling in a highly fertile agricultural soil. *ISME Comm* 2021;1:19. 10.1038/s43705-021-00020-4 PMC9723749 37938645

[73] Graf DRH, Jones CM, Zhao M. et al. Assembly of root-associated N2O-reducing communities of annual crops is governed by selection for *nosZ* clade I over clade II. *FEMS Microbiol Ecol* 2022;98:1–11. 10.1093/femsec/fiac092 PMC9397574 35927461

[74] Edgar RC . Search and clustering orders of magnitude faster than BLAST. *BMC Bioinformatics* 2021;26:2460–1. 10.1093/bioinformatics/btq461 20709691

[75] Caporaso JG, Kuczynski J, Stombaugh J. et al. QIIME allows analysis of high throughput community sequencing data. *Nat Methods* 2010;7:335–6. 10.1038/nmeth.f.303 20383131 PMC3156573

[76] Putkinen A, Siljanen HMP, Laihonen A. et al. New insight to the role of microbes in the methane exchange in trees: evidence from metagenomic sequencing. *New Phytol* 2021;231:524–36. 10.1111/nph.17365 33780002

[77] Manoharan L, Kushwaha SK, Ahrén D. et al. Agricultural land use determines functional genetic diversity of soil microbial communities. *Soil Biol Biochem* 2017;115:423–32. 10.1016/j.soilbio.2017.09.011

[78] Browne PD, Nielsen TK, Kot W. et al. GC bias affects genomic and metagenomic reconstructions, underrepresenting GC-poor organisms. *GigaScience* 2020;9:giaa008. 10.1093/gigascience/giaa008 32052832 PMC7016772

[79] Kozich JJ, Westcott SL, Baxter NT. et al. Development of a dual-index sequencing strategy and curation pipeline for analyzing amplicon sequence data on the MiSeq Illumina sequencing platform. *Appl Environ Microbiol* 2013;79:5112–20. 10.1128/AEM.01043-13 23793624 PMC3753973

[80] Glaze TD, Erler DV, Siljanen HMP. Microbially facilitated nitrogen cycling in tropical corals. *ISME J* 2021;16:68–77. 10.1038/s41396-021-01038-1 34226659 PMC8692614

[81] Marushchak ME, Kerttula J, Diáková K. et al. Thawing Yedoma permafrost is a neglected nitrous oxide source. *Nat Commun* 2021;12:7107. 10.1038/s41467-021-27386-2 34876586 PMC8651752

