# Correction to: Targeted metagenomics using probe capture detect a larger diversity of nitrogen and methane cycling genes in complex microbial communities than traditional metagenomics

**DOI:** 10.1093/ismeco/ycag020

**Published:** 2026-02-16

This is a correction to: Henri M P Siljanen, Lokeshwaran Manoharan, Angus S Hilts, Alexandre Bagnoud, Ricardo J E Alves, Christopher M Jones, Melina Kerou, Filipa L Sousa, Sara Hallin, Christina Biasi, Christa Schleper, Targeted metagenomics using probe capture detect a larger diversity of nitrogen and methane cycling genes in complex microbial communities than traditional metagenomics, *ISME Communications*, Volume 5, Issue 1, January 2025, https://doi.org/10.1093/ismeco/ycaf183

The name of the eighth co-author has been emended to read: “Filipa L. Sousa”.

The following has been added to the **Acknowledgements** section: “We thank PCI Microbiology, a community of researchers acting as associate editors, for taking on the peer review and recommendation (*https://doi.org/10.24072/pci.microbiol.100083*) of our preprint (*https://doi.org/10.1101/2022.11.04.515048*).”

