# Supplementary material for: Targeted metagenomics using probe capture detect a larger diversity of nitrogen and methane cycling genes in complex microbial communities than traditional metagenomics
Source: ISME Commun. 2025 Nov 1;5(1):ycaf183. doi: 10.1093/ismeco/ycaf183 (PMC12598625; doi:10.1093/ismeco/ycaf183)
Supplement: Supplementary_Fig_S1 [file supplementary_fig_s1.docx]

A)

B)

Fig. S1. The relative abundance of targeted functional genes originally mixed into the samples and in captured metagenomes retrieved with the custom HMMs searches. A) The relative abundance of each targeted functional gene in the original DNA mixture and that obtained by captured metagenomics (mean ± s.d., n=6) . Statistically significant differences between original and targeted metagenomic relative abundance according to pairwise comparisons with ANOVA are shown with asterisk (*P* < 0.05). B) Bars with different shades of grey show relative gene abundance values for each GC% category in the original community (O) and those obtained by targeted captured metagenomics (C).
