# Supplementary material for: Targeted metagenomics using probe capture detect a larger diversity of nitrogen and methane cycling genes in complex microbial communities than traditional metagenomics
Source: ISME Commun. 2025 Nov 1;5(1):ycaf183. doi: 10.1093/ismeco/ycaf183 (PMC12598625; doi:10.1093/ismeco/ycaf183)
Supplement: Supplementary_Fig_S2 [file supplementary_fig_s2.docx]

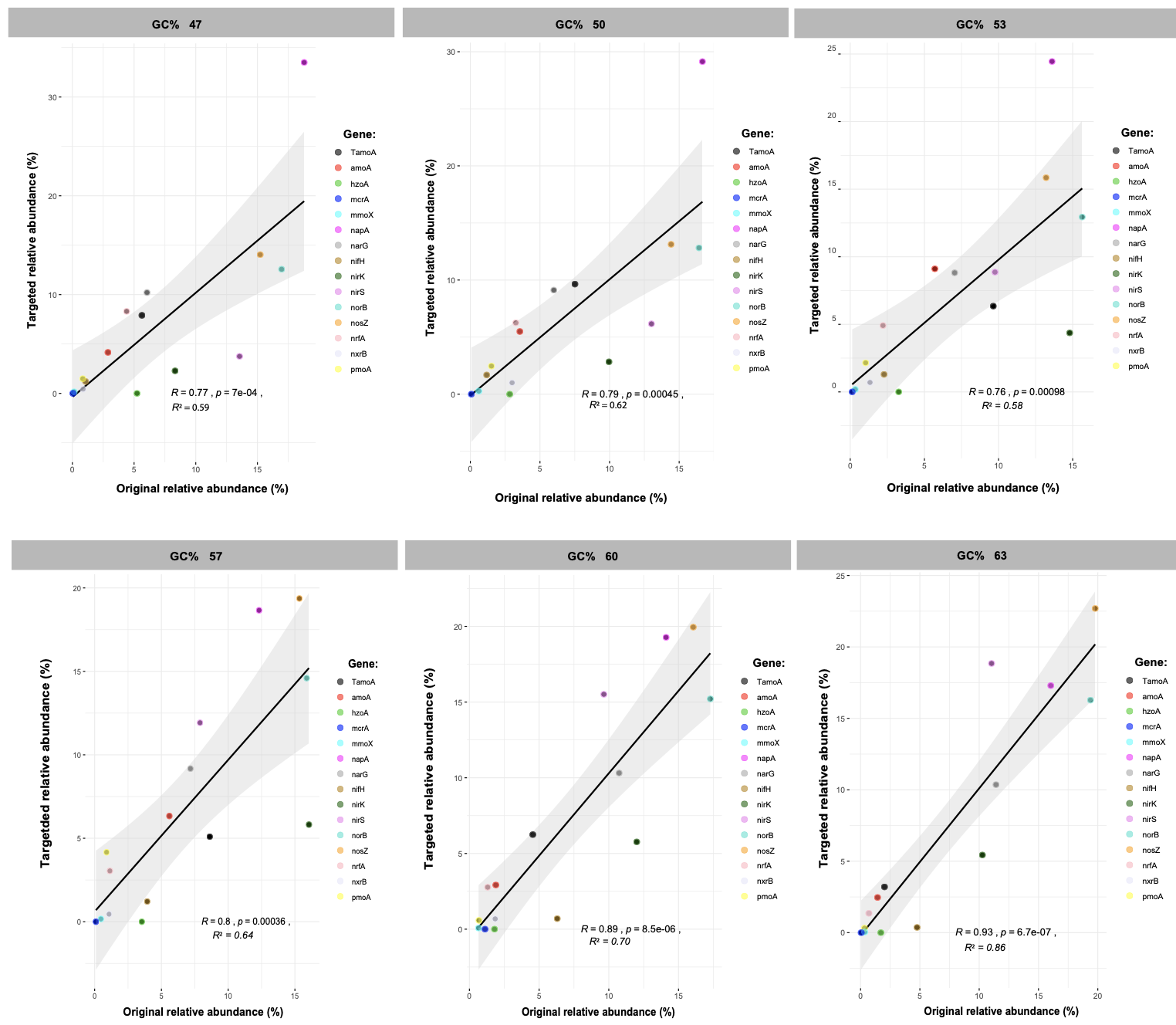


Fig. S2. Comparison of mock community between original (The relative abundance which was calculated with the amount of each functional gene and organism taken into the mock community and genome size.) and targeted metagenomics relative abundance produced for each different functional genes studied with probe hybridized targeted metagenomics for each GC% content separately.
