# Supplementary material for: Targeted metagenomics using probe capture detect a larger diversity of nitrogen and methane cycling genes in complex microbial communities than traditional metagenomics
Source: ISME Commun. 2025 Nov 1;5(1):ycaf183. doi: 10.1093/ismeco/ycaf183 (PMC12598625; doi:10.1093/ismeco/ycaf183)
Supplement: Supplementary_Fig_S3 [file supplementary_fig_s3.docx]

Fig. S3. Phylogenetic placement of *nosZ* reads obtained from agricultural soil using (n=3) A) shotgun metagenomics and B) targeted metagenomics (n=3). For illustration purposes all the replicas are pooled together. The reference phylogeny[12] is based on amino acid sequences analyzed using the LG+R10 substitution model in IQ-TREE, and node symbols indicate the location of placements in the reference tree. Symbol size corresponds to the relative abundance of reads placed at each node, and the scale bar indicates branch length in the reference tree. ‘Outgroup’ denotes distant homologues of *nosZ* with unknown function.
