# Supplementary material for: Targeted metagenomics using probe capture detect a larger diversity of nitrogen and methane cycling genes in complex microbial communities than traditional metagenomics
Source: ISME Commun. 2025 Nov 1;5(1):ycaf183. doi: 10.1093/ismeco/ycaf183 (PMC12598625; doi:10.1093/ismeco/ycaf183)
Supplement: Supplementary_Fig_S4 [file supplementary_fig_s4.docx]

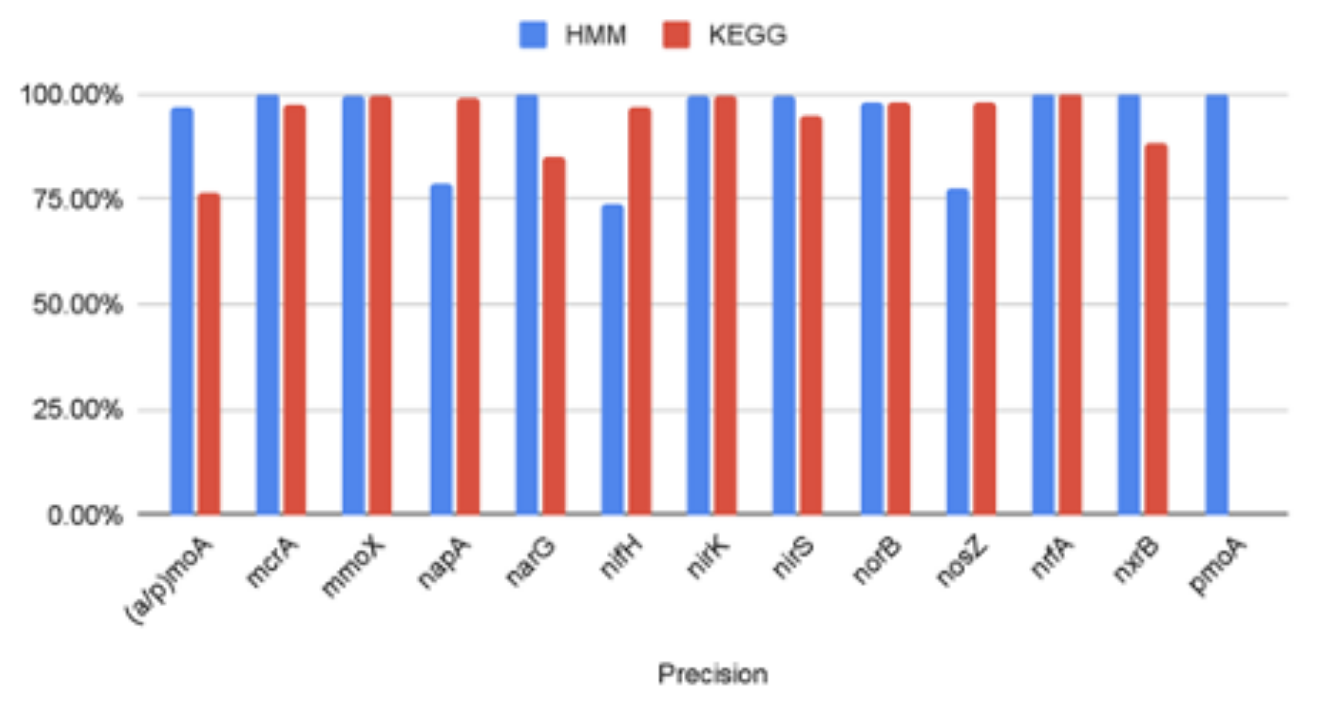

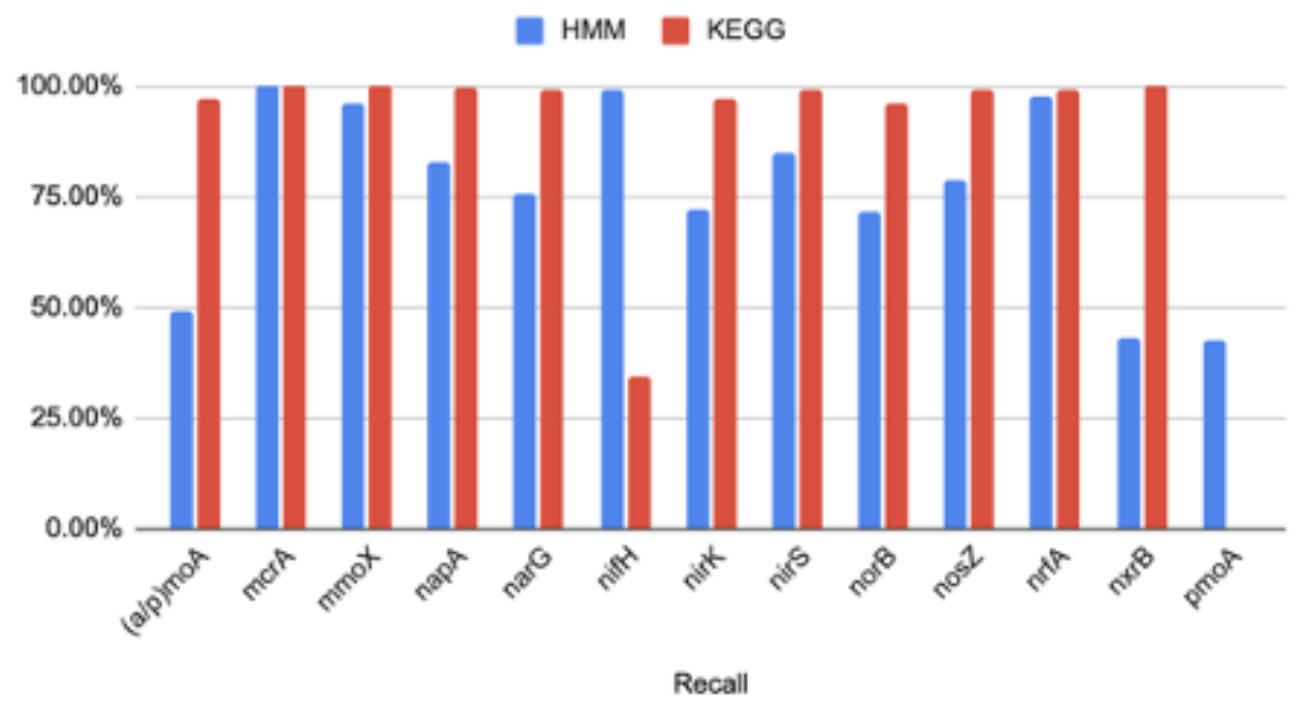
Fig. S4. Comparison of KEGG annotation and custom HMMs precision and recall for the mock-community comparison with different GC% categories combined.
