# Supplementary material for: Targeted metagenomics using probe capture detect a larger diversity of nitrogen and methane cycling genes in complex microbial communities than traditional metagenomics
Source: ISME Commun. 2025 Nov 1;5(1):ycaf183. doi: 10.1093/ismeco/ycaf183 (PMC12598625; doi:10.1093/ismeco/ycaf183)
Supplement: Supplementary_Fig_S5 [file supplementary_fig_s5.docx]

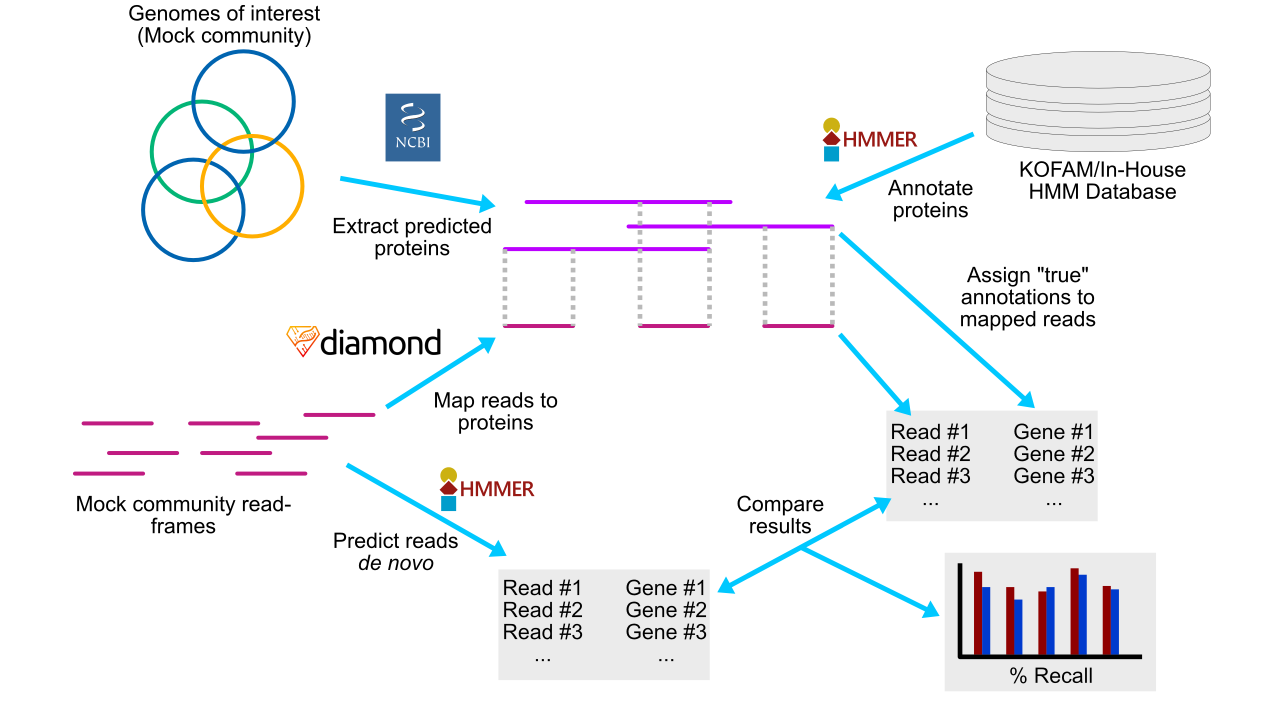


Fig. S5. Illustration of pipeline for determining TP, FP, TN, and FN de novo predictions by read mapping and HMM model prediction compared to genome predicted proteins.
