# Supplementary material for: Targeted metagenomics using probe capture detect a larger diversity of nitrogen and methane cycling genes in complex microbial communities than traditional metagenomics
Source: ISME Commun. 2025 Nov 1;5(1):ycaf183. doi: 10.1093/ismeco/ycaf183 (PMC12598625; doi:10.1093/ismeco/ycaf183)
Supplement: Supplementary_Table_S1 [file supplementary_table_s1.docx]

Table S1. The mock-community organisms genome accession, presence of the N and CH_4_ cycling genes in genome, median GC% of genome and DNA quality which was measured by for UV absorbance based on the with ratio of 260 [nm]/280 [nm] ratio.

| Organism: | genome accession (RefSeq) | Presence of N and CH_4_ genes | Median GC% of genome | A260/A280 |
| --- | --- | --- | --- | --- |
| *Nitrosospira multiformis* | [NC_007614.1](https://www.ncbi.nlm.nih.gov/nuccore/NC_007614.1) | *amoA*, *nirK*, *norB* | 53.3 | 2.03 |
| *Nitrososphaera viennensis* | CP007536.1 | *TamoA*, *nirK* | 52.07 | 2.07 |
| *Nitrospira defluvii* | NC_014355 | *nxrB* | 59 | 1.27 |
| *Ca.* Kuenenia stuttgartiensis*/hzoA PCR fragment from plasmid vector* |  | *hzoA* | 46.02 | 1.88 |
| *Pseudomonas aeruginosa PA96* | CP007224.1 | *narG*, *napA*, *nirS*, *norB*, *nosZ*-I | 66.2 | 2.11 |
| *Escherichia coli DH5a* | AE014075.1 | *nrfA*, *narG*, *napA* | 50.6 | 1.92 |
| *Shigella sonnei* strain FC1706 | CP014099.2 | *nrfA*, *napA* | 50.7 | 2.05 |
| *Cupriavidus metallidurans* CH34/CCUG 13724 | NC_007973.1 | *nosZ*-I, *nirS*, *norB*, *narG*, *napA* | 63.58 | 2.15 |
| *Cupriavidus necator* ATCC 17699 | NC_008313.1; NC_008314.1 | *nosZ*-I, *nirS*, *nor*, *narG*, *napA* | 66.3 | 2.16 |
| *Dyadobacter fermentans* DSM 18053 | NC_013037.1 | *nosZ*-II | 51.5 | 2.05 |
| *Pseudomonas stutzeri* JM300/DSM 10701 | NC_018177 | *napA*, *narG*, *nirS*, *nirK*, *norB*, *nosZ*-I | 63.3 | 2.17 |
| *Rhodobacter sphaeroides* DSM 158/ATCC 17023 | NC_007493.2; NC_007494.2 | *nifH*, *napA*, *nirK*, *norB*, *nosZ*-I | 68.77 | 2.16 |
| *Salinibacter ruber* DSM 13855 | NC_007677.1; NC_007678.1 | *nosZ*-II | 65.98 | 1.9 |
| *Sulfurimonas denitrificans* DSM 1251 | NC_007575.1 | *nosZ*-II, *nirS*, *norB*, *napA* | 34.5 | 2.13 |
| *Methylosinus trichosporium Ob3p* | CP023737.1 | *nifH*, 3x *pmoA*, *mmoX* | 65.82 | 1.42 |
| *Methylocella tundraea* | GCF_900749825.1 | *nifH*, *mmoX* | 63 | 1.11 |
| *Methylomicrobium buryatense 5B* | FO082060.1 | 1x*pmoA*, *norB* | 48.07 | 2.00 |
| *Methanoregula boonei* | NC_009712 | *nifH*, *mcrA* | 54.5 | 1.43 |
| *Methanolacinia petrolearia* | NC_014507 | *nifH*, *mcrA* | 47.4 |  |
