# Supplementary material for: Targeted metagenomics using probe capture detect a larger diversity of nitrogen and methane cycling genes in complex microbial communities than traditional metagenomics
Source: ISME Commun. 2025 Nov 1;5(1):ycaf183. doi: 10.1093/ismeco/ycaf183 (PMC12598625; doi:10.1093/ismeco/ycaf183)
Supplement: Supplementary_Table_S2 [file supplementary_table_s2.docx]

Table S2. The mock-community relative abundance composition, and weighted GC% content of each microorganisms and on each GC% category samples.

| **Organism:** | **GC 47% [Rel. Abun.]** | **GC 47% Weighted GC%** | **GC 50% [Rel. Abun.]** | **GC 50% Weighted GC%** | **GC 53% [Rel. Abun.]** | **GC 53% Weighted GC%** | **GC 57% [Rel. Abun.]** | **GC 57% Weighted GC%** | **GC 60% [Rel. Abun.]** | **GC 60% Weighted GC%** | **GC 63% [Rel. Abun.]** | **GC 63% Weighted GC%** |
| --- | --- | --- | --- | --- | --- | --- | --- | --- | --- | --- | --- | --- |
| *Nitrosospira multiformis* | 0.024 | 1.30 | 0.034 | 1.82 | 0.053 | 2.80 | 0.05 | 2.79 | 0.02 | 1.18 | 0.02 | 0.98 |
| *Nitrososphaera viennensis* | 0.143 | 7.43 | 0.22 | 11.27 | 0.267 | 13.88 | 0.24 | 12.60 | 0.16 | 8.31 | 0.08 | 4.04 |
| *Nitrospira defluvii* | 0.011 | 0.65 | 0.043 | 2.55 | 0.018 | 1.09 | 0.02 | 0.89 | 0.03 | 1.92 | 0.01 | 0.82 |
| *Ca.* Kuenenia stuttgartiensis/hzoA fragment in plasmid vector | 0.13 | 6.10 | 0.0 | 3.75 | 0.090 | 4.16 | 0.10 | 4.56 | 0.06 | 2.91 | 0.07 | 3.02 |
| *Pseudomonas aeruginosa* PA96 | 0.004 | 0.28 | 0.008 | 0.52 | 0.014 | 0.93 | 0.013 | 0.83 | 0.026 | 1.72 | 0.03 | 1.83 |
| *Escherichia coli* , DH5α | 0.096 | 4.84 | 0.084 | 4.28 | 0.048 | 2.44 | 0.023 | 1.14 | 0.041 | 2.05 | 0.02 | 1.15 |
| *Shigella sonnei* strain FC1706 | 0.016 | 0.79 | 0.010 | 0.49 | 0.013 | 0.65 | 0.009 | 0.48 | 0.005 | 0.28 | 0.004 | 0.21 |
| *Cupriavidus metallidurans* CH34/CCUG 13724 | 0.012 | 0.78 | 0.027 | 1.72 | 0.045 | 2.84 | 0.048 | 3.04 | 0.091 | 5.80 | 0.11 | 6.70 |
| *Cupriavidus necator* ATCC 17699 | 0.019 | 1.23 | 0.029 | 1.90 | 0.043 | 2.85 | 0.054 | 3.60 | 0.090 | 5.99 | 0.15 | 9.91 |
| *Dyadobacter fermentans* DSM 18053 | 0.17 | 8.78 | 0.068 | 3.50 | 0.114 | 5.86 | 0.099 | 5.08 | 0.054 | 2.80 | 0.02 | 1.02 |
| *Pseudomonas stutzeri* JM300/DSM 10701 | 0.022 | 1.42 | 0.025 | 1.58 | 0.045 | 2.82 | 0.064 | 4.05 | 0.129 | 8.16 | 0.13 | 8.46 |
| *Rhodobacter sphaeroides* DSM 158/ATCC 17023 | 0.021 | 1.46 | 0.011 | 0.79 | 0.045 | 3.12 | 0.092 | 6.34 | 0.111 | 7.61 | 0.17 | 11.35 |
| *Salinibacter ruber* DSM 13855 | 0.021 | 1.39 | 0.029 | 1.928 | 0.050 | 3.294 | 0.117 | 7.704 | 0.114 | 7.554 | 0.171 | 11.26 |
| *Sulfurimonas denitrificans* DSM 1251 | 0.26 | 9.85 | 0.287 | 9.889 | 0.123 | 4.260 | 0.043 | 1.475 | 0.002 | 0.073 | 0.008 | 0.28 |
| *Methylosinus trichosporium* Ob3p | 0.001 | 0.07 | 0.005 | 0.343 | 0.003 | 0.188 | 0.003 | 0.206 | 0.006 | 0.387 | 0.003 | 0.21 |
| *Methylocella tundraea* | 0.002 | 0.16 | 0.012 | 0.777 | 0.007 | 0.412 | 0.010 | 0.627 | 0.017 | 1.078 | 0.010 | 0.65 |
| *Methylomicrobium buryatense* 5B | 0.018 | 0.88 | 0.028 | 1.346 | 0.020 | 0.967 | 0.016 | 0.752 | 0.006 | 0.308 | 0.003 | 0.16 |
| *Methanoregula boonei* | 0.001 | 0.028 | 0.001 | 0.059 | 0.002 | 0.103 | 0.001 | 0.050 | 0.019 | 1.039 | 0.001 | 0.06 |
| *Methanolacinia petrolearia* | 0.000 | 0.011 | 0.001 | 0.024 | 0.001 | 0.042 | 0.001 | 0.040 | 0.010 | 0.479 | 0.000 | 0.02 |
| sum: | 1.00 | 47.43 | 1.00 | 48.51 | 1.00 | 52.70 | 1.00 | 56.26 | 1.00 | 59.64 | 1.00 | 62.10 |
