# Supplementary material for: Targeted metagenomics using probe capture detect a larger diversity of nitrogen and methane cycling genes in complex microbial communities than traditional metagenomics
Source: ISME Commun. 2025 Nov 1;5(1):ycaf183. doi: 10.1093/ismeco/ycaf183 (PMC12598625; doi:10.1093/ismeco/ycaf183)
Supplement: Supplementary_Table_S3 [file supplementary_table_s3.docx]

Table S3. Soil properties of used samples in the shotgun vs. targeted analysis and targeted vs. archaeal *amoA* amplicon analysis.

| Site | Coordinates | Corg/Norg | pH H_2_O | NH_4_^+^ [μg NH_4_^+^ -N·g -1 dry soil] | NO_2_^-^ [μg NO_2_^-^ -N·g -1 dry soil] | NO_3_^-^ [μg NO_3_^-^ -N·g -1 dry soil] | Fe II [μmol·g -1 dry soil] | Fe III [μmol·g -1 dry soil] | Ref.: |
| --- | --- | --- | --- | --- | --- | --- | --- | --- | --- |
| Agricultural field, Hungary^#~~&~~^ | 47°31N 16°59E | 17.9±5.3 | 6.7±0.16 | 0.02±0.004 | 0.23±0.1 | 3.4±1.9 | 1.5±0.07 | 43.3±4.8 | This study |
| Belfontaine wetland, France^#^ | 46°34N 6°04E | 14±0.6 | 6.9±0.1 | 0.04±0 | 0.04±0 | 0.002±0.001 | 78.5 ± 43.9 | 78.5 ± 43.9 | [22] |

bd = below detection limit

NA = not analyzed

^#^= shotgun vs. targeted comparison, Fig. 2,3.

^&^= Targeted vs. *amoA* amplicon analysis, Fig. 4, S3.
