# Supplementary material for: Targeted metagenomics using probe capture detect a larger diversity of nitrogen and methane cycling genes in complex microbial communities than traditional metagenomics
Source: ISME Commun. 2025 Nov 1;5(1):ycaf183. doi: 10.1093/ismeco/ycaf183 (PMC12598625; doi:10.1093/ismeco/ycaf183)
Supplement: Supplementary_Table_S4 [file supplementary_table_s4.docx]

Table S4. Number of total sequences produced, targeted reads and recovery of targeted sequences after capture and depth of the sequencing.

| Sample: | Sequencing method | Total reads | | Targeted reads | % Targeted from total | Depth, of bases: | |
| --- | --- | --- | --- | --- | --- | --- | --- |
| Mock community GC 47% | Targeted metagenomics | 1844716 | 1128755 | | 61.2 | 1.0G |  |
| Mock community GC 50% | Targeted metagenomics | 2281740 | 1437919 | | 63.0 | 1.3G |  |
| Mock community GC 53% | Targeted metagenomics | 2006493 | 1360701 | | 67.8 | 1.1G |  |
| Mock community GC 57% | Targeted metagenomics | 2346193 | 1692037 | | 72.1 | 1.3G |  |
| Mock community GC 60% | Targeted metagenomics | 2625812 | 1771389 | | 67.5 | 1.5G |  |
| Mock community GC 63% | Targeted metagenomics | 2429736 | 1640046 | | 67.5 | 1.3G |  |
| 44 FR_Bellfonte_wetland_BF_3_8 | Targeted metagenomics | 214947 | 124886 | | 58.1 | 114.4M |  |
| 50 FR_Bellfonte_wetland_BF_10-1 | Targeted metagenomics | 285234 | 161399 | | 56.6 | 147.5M |  |
| 51 FR_Bellfonte_wetland_BF_10-2 | Targeted metagenomics | 198756 | 109938 | | 55.3 | 104.1M |  |
| 47 HG_agricultural_soil_H16 | Targeted metagenomics | 246209 | 130449 | | 53.0 | 132M |  |
| 48 HG_agricultural_soil_H17 | Targeted metagenomics | 220005 | 116976 | | 53.2 | 117.5M |  |
| 49 HG_agricultural_soil_H18 | Targeted metagenomics | 311535 | 162396 | | 52.1 | 160.7M |  |
|  |  |  |  | |  |  |  |
| Bellfonte_wetland BF_3-8 | Shotgun metagenomics | 21464245 | 5102 | | 0.024 | 6.4G |  |
| Bellfonte_wetland BF_10-1 | Shotgun metagenomics | 43620482 | 6434 | | 0.015 | 12.9G |  |
| Bellfonte_wetland BF_10-2 | Shotgun metagenomics | 50203331 | 6865 | | 0.014 | 14.9G |  |
| agricultural_soil H16 | Shotgun metagenomics | 53422156 | 3457 | | 0.007 | 16G |  |
| agricultural_soil H17 | Shotgun metagenomics | 22140941 | 2883 | | 0.013 | 6.6G |  |
| agricultural_soil H18 | Shotgun metagenomics | 57814232 | 4213 | | 0.007 | 17.3G |  |
|  |  |  |  | |  |  |  |
| Agricultural H16 | Amplicon sequencing | 58006 | 49679 | | 85.64 | 31M |  |
| Agricultural H17 | Amplicon sequencing | 27565 | 23648 | | 85.79 | 14.9M |  |
| Agricultural H18 | Amplicon sequencing | 35955 | 30830 | | 85.75 | 19.3M |  |
