# Supplementary material for: Targeted metagenomics using probe capture detect a larger diversity of nitrogen and methane cycling genes in complex microbial communities than traditional metagenomics
Source: ISME Commun. 2025 Nov 1;5(1):ycaf183. doi: 10.1093/ismeco/ycaf183 (PMC12598625; doi:10.1093/ismeco/ycaf183)
Supplement: Siljanen_etal_2025_ycaf183_Supplementary_Materials [file siljanen_etal_2025_ycaf183_supplementary_materials.docx]

**Supplementary materials for,**

**Targeted metagenomics using probe capture detects a larger diversity of nitrogen and methane cycling genes in complex microbial communities than traditional metagenomics**

Henri M.P. Siljanen^1,2^, Lokesh Manoharan^2a^, Angus S. Hilts^2^, Alexandre Bagnoud^2b^, Ricardo J.E. Alves^2c^, Christopher M. Jones^3^, Melina Kerou^2^, Felipa L. Sousa^2^, Sara Hallin^3^, Christina Biasi^1,4^, Christa Schleper^2^

1. Department of Environmental and Biological Sciences, University of Eastern Finland, Kuopio, Finland

2. Department of Functional and Evolutionary Ecology, University of Vienna, Vienna, Austria

3. Department of Forest Mycology and Plant Pathology, Swedish University of Agricultural Sciences, Uppsala, Sweden.

4. Department of Ecology, University of Innsbruck, Innsbruck, 6020, Austria

Present address:

1. National Bioinformatics Infrastructure Sweden (NBIS), SciLifeLab, Department of Laboratory Medicine, Lund University, Lund, Sweden.
2. Membratec S, Ecoprac de Daval C 1, CH-3960 Sierre, Switzerland.

Climate and Ecosystem Sciences Division, Lawrence Berkeley National Laboratory, Berkeley, CA, USA

**Supplementary Material and Methods**

**Targeted metagenomic library preparation, target enrichment of libraries and sequencing with Illumina Miseq**

To prepare DNA for targeted metagenomics with probe capture, DNA was first fragmented and indexed as follows. For each sample, sequencing indexes and sequencing adapters were provided as commercial service by the Centre for Genomic Research (CGR) -laboratories, at the University of Liverpool, Liverpool, UK. Libraries were produced with KAPA HyperPlus Library Preparation (Roche) kit to produce insert sizes of 630 bp according to the manufacturer’s instructions. The protocol is shown in Fig. 1. and described Supplementary Material and Methods. 150ng of environmental DNA was fragmented with KAPA fragmentation buffer and KAPA Frag Enzyme at +37°C for 20min. Fragmented DNA was ligated to SeqCap Adapters A and B, +20°C for 15 minutes. Fragments were purified 0.65x volumes of AMPure XP Reagent for 5 min at room temperature in magnetic particle collector (MPC). Then the clear supernatant was removed and the library was washed twice with 200 μl of 80% ethanol. The libraries were removed from MPC by eluting them to 53 μl of elution buffer (10 mM Tris-HCl, pH 8.0) by incubated two minutes. Each library was amplified for 7 cycles, with KAPA HyperPlus Library Preparation kit in order to add the indexes for each sample (cycling conditions: +98°C, 15 sec; 60° for 30 sec; +72°C for 30 sec). Amplified libraries were purified with AMPure XP beads as above.

The hybridization reactions to the NimbleGen SeqCap EZ Developer Probe set were performed according to the manufacturer’s instructions. The hybridization was done in non-binding DNA background 25 μg, on top of 1 μg of each library equally pooled Multiplexed DNA Sample Library were added. The Multiplexed Hybridization Enhancing Oligo Pool (2,000 pmol in 2μl volume), the SeqCap HE Universal Oligo (1,000 pmol in 1μl volume) and (1,000 pmol in 1μl volume) SeqCap HE Index Oligo Pool) was mixed with 1 μg of equally pooled Multiplexed DNA Sample Library. Two volumes of AMPure XP Reagent was added on top of the above mixture and thoroughly mixed. Samples were incubated for 10 minutes to allow the sample library to bind to the beads. Samples were placed on the MPC to capture the beads and the solution allowed to clear. Once clear, the supernatant was discarded carefully, to not disturb the beads. Afterwards 190 μl of 80% ethanol was added to the samples containing the bead-bound DNA samples. The samples were left on the MPC during this step. Samples were incubated at room temperature for >30 seconds. The 80% ethanol was carefully removed and discarded, without disturbing the beads. Beads were dried at room temperature with the tube lid open for 5 minutes (or until dry).

A master mix of the following reagents was prepared, scaling up to reflect the number of captures: 7.5 μl of the 2x Hybridization Buffer, 3 μl of Hybridization Component A. Then 10.5 μL of the Hybridization Buffer/Hybridization Component A mix from the previous step was added to the bead-bound DNA sample. Samples were removed from the MPC and mixed thoroughly. It was important that enough mixing was performed at this step to yield a homogeneous mixture. It was left to sit at room temperature for 2 minutes. Sampled were then placed on the MPC. After liquid cleared, 10.5 μL of supernatant (entire volume) was removed and placed in a new tube containing 4.5 μl of the SeqCap EZ Developer Probe pool. This was mixed thoroughly. Hybridization incubation was performed in a thermocycler using the following program with heated lid set to 10 °C above the block temperature: incubation was 95°C for 5min, and 47°C for 72 hours (SeqCap EZ HyperCap Workflow protocol [1]).

To purify the hybridized libraries the SeqCap Pure Capture Bead Kit was used. To prepare the beads for binding the samples, 50 μl of beads (streptavidin coated magnetic beads) was added to the 1.5 ml tube at room temperature for capturing reaction. Tubes were placed on a MPC, the supernatant removed and beads were washed two times with 100 μl of 1x Bead Wash Buffer. Afterwards it was removed from the MPC and mixed by pipetting up and down. Beads were bound on the magnetic particle collector. Once the liquid was clear, the supernatant was removed. In the second wash, the beads were aliquoted with 50 μl of 1X Bead Washing Buffer. Beads were bound and the supernatant again removed. One hybridization sample (15 μl volume) was added to the washed SeqCap Pure Capture Beads. Samples were bound to the beads by placing the samples in a thermocycler at +47°C for 15 minutes (heated lid at +57°C). Then 100 μl of 1x Wash Buffer was added on top of the Capture Beads and bead-bound DNA and mixed thoroughly. The tube was placed on the MPC to capture the beads. The solution was allowed to clear, and the supernatant removed, while being careful not to disturb the beads. Afterwards, 200 μl of 1x Stringent Wash Buffer was added to each capture reaction. Those were mixed by pipetting up and down, when tubes were not in a MPC. Samples were placed in the thermocycler to +47°C for 5 minutes. The wash was repeated with 1x Stringent Wash Buffer. Next, 200 μl of 1x Wash Buffer I was added, vortexed for 10 second, and incubated 1 minute at room temperature. Once the solution was clear, it was removed, again being careful not to disturb the beads. The washing with 1X Wash buffer II was repeated, and then 1x with Washing Buffer III. Samples were removed from the MPC and 53 μl of PCR-grade water was added to the bead-bound sample. Sampled were then incubated at room temperature for 2 minutes and placed in the MPC. Then 50 μl of clear solution was collected in a new tube.

Sequencing for probe hybridized and washed DNA was performed with Illumina MiSeq PE300 chemistry in the Centre for Genomic Research (CGR), University of Liverpool, Liverpool, UK, resulting in 198,700-311,500 reads per sample for the environmental samples and up to 2,600,000 reads for the mock communities.

**Annotation of coding sequences from the mock community dataset**

Predicted proteomes from the mock communities were retrieved from NCBI to count the number of each functional genes per genome. For *Nitrospira defluvii*; NC_014355.1, genes were predicted from the genome nucleotide sequence using Prodigal (v2.6.3)[2]. Proteins were annotated using KOFAM (HMM database of KEGG Orthologs (KO)). Each protein was assigned the top hitting KO. Where available, cut-offs provided with KOFAMscan[3] were used to filter spurious results.

**Evaluation of functional annotation of reads**

Protein predictions for each read were performed using nhmmer from the HMMER suite (v.3.3)[4] using the in-house generated HMMs for target genes as well as KOFAM as described above. An inclusion threshold of <0.0001 was used when assigning protein predictions for the reads. For a given read, using all six of the translated frames, up to four protein assignments could be made, evaluated based on KO bit scores or identity filtering. Reads assigned KOs for the function of interest were counted as positively identified hits. Subsequently, reads were mapped to their corresponding coding sequences (CDS) from the mock community organisms to determine which reads constituted “true positives” (TP) or “true negatives” (TN). Reads mapped to a given CDS were assigned functions according to the above-mentioned KOFAM CDS annotation as their “true function”. If the HMM assignment (both in-house and from KOFAM) of a read matched the functional assignment of the CDS to which it was mapped, then it was counted as a TP. The TP, false positive (FP), and false negative (FN) values were summed up for all models using all methods, and precision and recall were calculated. Read mapping (described above) was used to determine the total number of possible TPs of the reads (Fig. S6), from which the actual number of TPs was subtracted to calculate the number of FNs. Precision was calculated as the ratio of TPs to total positive detections (i.e., number of TPs and FPs). Recall was calculated as the ratio of TPs to all theoretically obtainable true positives (i.e., sum of TPs and FNs). The script is available on Zenodo[5] /probe-capture/tree/main/3-other_scripts/Angus_scripts -folder.

**Processing of amplicon *amoA* reads**

Archaeal *amoA* (Thaumarchaeal-*amoA*, *TamoA*) amplicon reads processing was carried out by a custom-based Python script available at Zenodo[5] [/probe-capture/tree/main](https://github.com/alex-bagnoud/probe-capture/tree/main)/1-scripts/1-amplicon_seq_script_v1.sh -folder. Briefly, quality trimming of sequencing reads was performed by truncating amplicon forward read R1 reads to 200bp and reverse read R2 180bp, discarding all reads with an expected error greater than 0.5. The *amoA* reads were dereplicated and amplicon sequence variants (ASVs) were assigned to taxonomic bins described in the reference *amoA* gene database [6] using a 55% identity cutoff with USEARCH8[7]. Chimeras were filtered out with UCHIME[8]. For the remaining ASVs, quality controlled R1 and R2 reads were fused together, classified with UCLUST in QIIME[9] and clustered to OTUs with 97% sequence identity cut-off for each taxonomic bins. Taxonomic bins of each *TamoA* gene cluster were generated using the reference gene database and taxonomy by[6][10,11]. Relative abundance of *TamoA* taxonomic bins was calculated as follows: Relative abundance of *TamoA* taxonomic bin x = (Abundance of *TamoA* taxonomix bin x / total *TamoA* reads) x 100. In Fig. 4A, the relative abundance of each taxonomic bin (relative to the column sum) was plotted for each sample. The sequencing depth for the *TamoA* gene amplicon library was ~27,000-58,000 PE250 reads per sample up to 31Mb for the agricultural soil (Table S4).

**Processing of metagenomic *amoA* reads**

Archaeal *amoA* (Thaumarchaeal-*amoA*, *TamoA*) reads from targeted- and shotgun metagenomics, were identified with the *TamoA* in-house HMM model. The forward reads were dereplicated and assigned to the reference *amoA* gene database using a 55% identity cutoff with USEARCH8[7]. Chimeras were filtered out with UCHIME[8], all reads were clustered with UCLUST with 97% sequence identity in QIIME and assigned to taxonomic bins based on the closest *amoA* genes from the taxonomy database of OTUs[6,9]. Relative abundance was calculated as follows: Relative abundance of *TamoA* taxonomic bin x = (Abundance of *TamoA* taxonomix bin x / total *TamoA* reads) x 100. In the targeted metagenomics approach, multiple detection of the same taxonomic bin is possible because of six probes are used for each sequence cluster. However, when relative abundance for each detected taxonomic bin is calculated for targeted metagenomic, if multiple detection occurs in the relative abundance calculation the community composition is balanced because of equal number of probes per each sequence cluster. Therefore, the relative abundance of different sequencing methods can be compared. The sequencing depth for the *TamoA* gene for three shotgun metagenomics *TamoA* reads out of ~22-58M PE150 reads per sample up to 17 Gb, and for targeted metagenomics 4472-8230 *TamoA* reads out of ~220-311k PE300 reads per sample up to 160 Mb, for the agricultural soil (Table S4). The metagenomic reads processing is described in the custom-made script available at Zenodo[5] for [probe-capture: tree/main](https://github.com/alex-bagnoud/probe-capture/tree/main)/1-scripts/2-probe_capture_script_v1.sh -folder, and [shotgun metagenomics: tree/main](https://github.com/alex-bagnoud/probe-capture/tree/main)/1-scripts/3-metagenomics_script_v1.sh -folder.

**Phylogenetic placement analysis of *nosZ* reads from shotgun and targeted metagenomics libraries.**

A reference alignment and phylogeny for *nosZ* was generated from full length *nosZ* amino acid sequences obtained from genomes downloaded from the NCBI genome database (accessed October 2019). A profile HMM for *nosZ* was generated from a previously published *nosZ* dataset, as described above[12]. Reads were converted to amino acid sequences and then aligned using *hmmalign* within the HMMer suite (v.3.1.2)[4], and the homologous sections were identified using FastTree (v. 2.1.11)[13]. The reference protein alignment was used for phylogenetic reconstruction using IQ-TREE (v1.6.12)[14] with best model selection and 1000 ultrafast bootstraps. The Le-Gascuel (LG) substitution model with 10 rate categories was selected after automatic model selection. For the phylogenetic placement of *nosZ* reads obtained using either shotgun metagenome sequencing (n=3) or targeted metagenomics (n=3) of the agricultural site, the relevant reads were pooled together and clustered with CD-HIT[15] at 90% sequence identity before translating and aligning to the reference *nosZ* alignment using *hmmalign*. The translated reads were then placed in the reference phylogeny using the next generation evolutionary placement algorithm (EPA-NG v0.3.8;[16]), with the same model parameters as used for constructing the reference phylogeny. The placement positions with the highest likelihood weight ratio were plotted on the reference phylogeny in R using the ‘ggtree’ package (v3.4.2,[17]). Script available for *nosZ* phylogenetic placements at Zenodo[5] /probe-capture/tree/main/3-other_scripts/Chris_Jones_scripts -folder.

**Supplementary Results**

**Precision of gene identification in mock communities**

Precision for the in-house HMMs ranged from 74.1% (*nifH*) to 100% (*mcrA*) (Fig. S5). The average precision of the models was 93.4%, with a median of 99.8% (excluding *amoA* and *pmoA*). The recall ranged from 42.8% (*nxrB*) to 100% (*mcrA*). The average recall was 81.9%, with a median recall of 82.8%. Because KEGG only provides a single model covering the homologous genes *pmoA* and *amoA*, it was not possible to accurately calculate the precision and recall for these genes individually. A precision estimate could still be determined for the two genes, where a “true positive” is defined as a hit for a read identified by the in-house models as *amoA* or *pmoA* mapped to a CDS identified by the HMM for (*a*/*p*)*moA* genes provided by KEGG (K10944). In this case, the precision values for the *amoA* and *pmoA* genes were estimated to be 96.9%, and 100%, respectively. It is important to note that these estimates do not exclude the possibility that the in-house models misclassified reads belonging to these homologous protein families.

Precision and recall values were similar using KOFAMs instead of in-house HMMs. However, the results for *amoA* and *pmoA* genes were combined due to a single model for the associated KO for both genes, as mentioned above. Precision ranged from 76.5% (*amoA*/*pmoA*) to 100.0% (*mcrA*), while recall varied from 34.3% (*nifH*) to 100% (*mcrA* and *nxrB*). The average and median precision values were 94.5% and 97.7%, while the average and median recall values were 93.39% and 99.03%, respectively. The precision of KOs was lower for *napA* than for other genes when compared with custom DNA HMMs. This could be accounted for by the presence of formate dehydrogenase, a known homolog of *napA*. Formate dehydrogenase (K00123) was indeed identified among the genomes used for the mock community, so it is possible that the custom HMM was misclassifying reads originating from this known ortholog to *napA*[18].

**Supplementary Discussion**

Our custom HMM models could assign reads to the correct genomes based on full protein sequences with higher precision than models based on KEGG Orthologs, especially when used to predict putative functions from short DNA fragments, such as metagenomic reads. This accuracy indicates that the HMMs can reliably classify reads in the mock community and are sensitive enough to detect distantly related genes or novel gene variants. Notably, reads identified as false positives may not actually be incorrect but could represent novel gene variants not captured by KEGG Orthologs.

Moreover, variation in detection efficiency may also result from differences in probe hybridization. For instance, probes with higher GC% content are expected to bind more strongly to their targets given the higher binding energy between guanine and cytosine than between adenine and thymine. Although our approach successfully identified *hzoA* genes in the environmental samples, it failed to detect them in mock communities. It is possible that this was due to the PCR-generated genomic fragment used as source material for *hzoA* genes in the mock community, which, unlike other target genes provided in the whole genomic DNA, is more susceptible to degradation or structure-related constraints on probe hybridization.

Despite the technological advances and decline in cost of high-throughput sequencing, shotgun metagenomics remain impractical and prohibitively expensive to capture the diversity of specific, low abundant functional groups in complex environments, such as soils and sediments, especially in longitudinal studies. The current cost of generating the probes for targeted metagenomics is about 20-50 € per sample depending on the probe manufacturer. If the goal is to have a focused, comprehensive view of the diversity of functional guilds involved in inorganic nitrogen or methane cycling in a certain ecosystem, then targeted metagenomics can circumvent the high cost and overabundance of data generated by shotgun metagenomics, as well as provide more quantitative data and more information on the diversity of the genes of interest. In turn, PCR-based assays are limited by the biases associated with any set of primers targeting a large diversity of sequences, assay efficiencies which preclude the detection of highly divergent new sequence diversity as well as known issues with the generation of chimeric sequences during amplification. The targeted metagenomics approach circumvents these limitations by using multiple short probes targeting different regions of each gene, which largely increases the likelihood of capturing sequence variants that elude PCR amplification. Thus, this approach has not only the potential to capture rare and novel gene diversity in complex environments, but also to identify cryptic microorganisms in low-biomass samples or involved in suggested CH_4_ metabolisms, such as in the tree phyllosphere[19] and nitrogen cycling in coral holobiont[20]. Moreover, targeted metagenomics can also overcome issues associated with running and comparing multiple independent PCR assays when investigating several distinct targets. In that sense, this approach effectively represents a PCR-independent, multiplex approach to characterize simultaneously and in-depth the distributions of a broad range of functional genes, providing a holistic view of the status of the nitrogen and methane cycles in the studied ecosystems. This is especially advantageous when combined with functional studies, such as the determination of N-transformation rates and *in situ* fluxes, as showcased by a study of N_2_O emissions in thawing Yedoma permafrost sites over time[21]. In this study, the application of targeted metagenomics with the N-cycling probe dataset presented here revealed that changes in the N-cycling microbial community composition were responsible for an increase in N_2_O emissions in revegetated Yedoma soils, which had undergone thawing a decade prior.

**Supplementary figures,**

A)

B)

Fig. S1. The relative abundance of targeted functional genes originally mixed into the samples and in captured metagenomes retrieved with the custom HMMs searches. A) The relative abundance of each targeted functional gene in the original DNA mixture and that obtained by captured metagenomics (mean ± s.d., n=6) . Statistically significant differences between original and targeted metagenomic relative abundance according to pairwise comparisons with ANOVA are shown with asterisk (*P* < 0.05). B) Bars with different shades of grey show relative gene abundance values for each GC% category in the original community (O) and those obtained by targeted captured metagenomics (C).


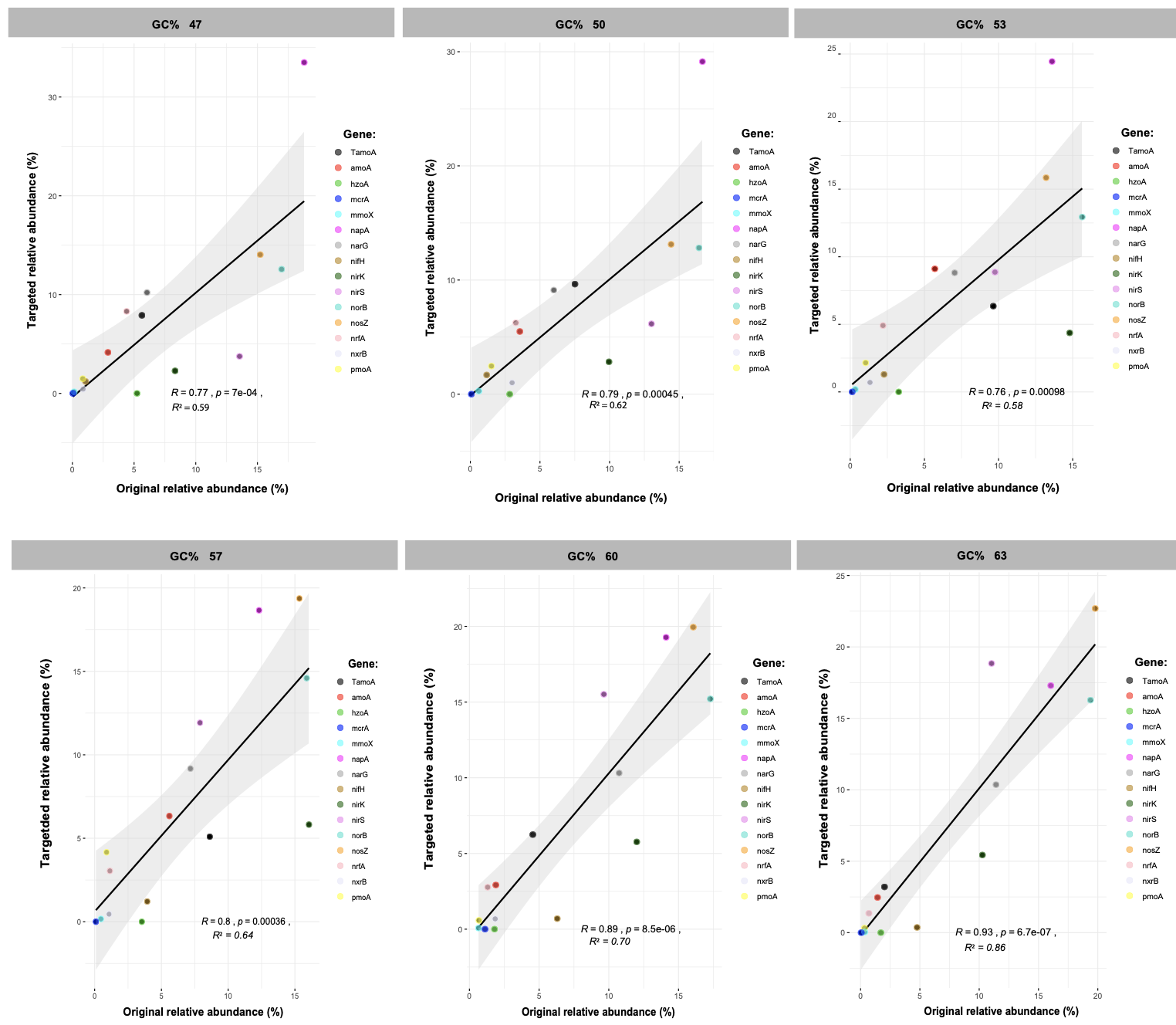


Fig. S2. Comparison of mock community between original (The relative abundance which was calculated with the amount of each functional gene and organism taken into the mock community and genome size.) and targeted metagenomics relative abundance produced for each different functional genes studied with probe hybridized targeted metagenomics for each GC% content separately.

Table S1. The mock-community organisms genome accession, presence of the N and CH_4_ cycling genes in genome, median GC% of genome and DNA quality which was measured by for UV absorbance based on the with ratio of 260 [nm]/280 [nm] ratio.

| Organism: | genome accession (RefSeq) | Presence of N and CH_4_ genes | Median GC% of genome | A260/A280 |
| --- | --- | --- | --- | --- |
| *Nitrosospira multiformis* | [NC_007614.1](https://www.ncbi.nlm.nih.gov/nuccore/NC_007614.1) | *amoA*, *nirK*, *norB* | 53.3 | 2.03 |
| *Nitrososphaera viennensis* | CP007536.1 | *TamoA*, *nirK* | 52.07 | 2.07 |
| *Nitrospira defluvii* | NC_014355 | *nxrB* | 59 | 1.27 |
| *Ca.* Kuenenia stuttgartiensis*/hzoA PCR fragment from plasmid vector* |  | *hzoA* | 46.02 | 1.88 |
| *Pseudomonas aeruginosa PA96* | CP007224.1 | *narG*, *napA*, *nirS*, *norB*, *nosZ*-I | 66.2 | 2.11 |
| *Escherichia coli DH5a* | AE014075.1 | *nrfA*, *narG*, *napA* | 50.6 | 1.92 |
| *Shigella sonnei* strain FC1706 | CP014099.2 | *nrfA*, *napA* | 50.7 | 2.05 |
| *Cupriavidus metallidurans* CH34/CCUG 13724 | NC_007973.1 | *nosZ*-I, *nirS*, *norB*, *narG*, *napA* | 63.58 | 2.15 |
| *Cupriavidus necator* ATCC 17699 | NC_008313.1; NC_008314.1 | *nosZ*-I, *nirS*, *nor*, *narG*, *napA* | 66.3 | 2.16 |
| *Dyadobacter fermentans* DSM 18053 | NC_013037.1 | *nosZ*-II | 51.5 | 2.05 |
| *Pseudomonas stutzeri* JM300/DSM 10701 | NC_018177 | *napA*, *narG*, *nirS*, *nirK*, *norB*, *nosZ*-I | 63.3 | 2.17 |
| *Rhodobacter sphaeroides* DSM 158/ATCC 17023 | NC_007493.2; NC_007494.2 | *nifH*, *napA*, *nirK*, *norB*, *nosZ*-I | 68.77 | 2.16 |
| *Salinibacter ruber* DSM 13855 | NC_007677.1; NC_007678.1 | *nosZ*-II | 65.98 | 1.9 |
| *Sulfurimonas denitrificans* DSM 1251 | NC_007575.1 | *nosZ*-II, *nirS*, *norB*, *napA* | 34.5 | 2.13 |
| *Methylosinus trichosporium Ob3p* | CP023737.1 | *nifH*, 3x *pmoA*, *mmoX* | 65.82 | 1.42 |
| *Methylocella tundraea* | GCF_900749825.1 | *nifH*, *mmoX* | 63 | 1.11 |
| *Methylomicrobium buryatense 5B* | FO082060.1 | 1x*pmoA*, *norB* | 48.07 | 2.00 |
| *Methanoregula boonei* | NC_009712 | *nifH*, *mcrA* | 54.5 | 1.43 |
| *Methanolacinia petrolearia* | NC_014507 | *nifH*, *mcrA* | 47.4 |  |

Table S2. The mock-community relative abundance composition, and weighted GC% content of each microorganisms and on each GC% category samples.

| **Organism:** | **GC 47% [Rel. Abun.]** | **GC 47% Weighted GC%** | **GC 50% [Rel. Abun.]** | **GC 50% Weighted GC%** | **GC 53% [Rel. Abun.]** | **GC 53% Weighted GC%** | **GC 57% [Rel. Abun.]** | **GC 57% Weighted GC%** | **GC 60% [Rel. Abun.]** | **GC 60% Weighted GC%** | **GC 63% [Rel. Abun.]** | **GC 63% Weighted GC%** |
| --- | --- | --- | --- | --- | --- | --- | --- | --- | --- | --- | --- | --- |
| *Nitrosospira multiformis* | 0.024 | 1.30 | 0.034 | 1.82 | 0.053 | 2.80 | 0.05 | 2.79 | 0.02 | 1.18 | 0.02 | 0.98 |
| *Nitrososphaera viennensis* | 0.143 | 7.43 | 0.22 | 11.27 | 0.267 | 13.88 | 0.24 | 12.60 | 0.16 | 8.31 | 0.08 | 4.04 |
| *Nitrospira defluvii* | 0.011 | 0.65 | 0.043 | 2.55 | 0.018 | 1.09 | 0.02 | 0.89 | 0.03 | 1.92 | 0.01 | 0.82 |
| *Ca.* Kuenenia stuttgartiensis/hzoA fragment in plasmid vector | 0.13 | 6.10 | 0.0 | 3.75 | 0.090 | 4.16 | 0.10 | 4.56 | 0.06 | 2.91 | 0.07 | 3.02 |
| *Pseudomonas aeruginosa* PA96 | 0.004 | 0.28 | 0.008 | 0.52 | 0.014 | 0.93 | 0.013 | 0.83 | 0.026 | 1.72 | 0.03 | 1.83 |
| *Escherichia coli* , DH5α | 0.096 | 4.84 | 0.084 | 4.28 | 0.048 | 2.44 | 0.023 | 1.14 | 0.041 | 2.05 | 0.02 | 1.15 |
| *Shigella sonnei* strain FC1706 | 0.016 | 0.79 | 0.010 | 0.49 | 0.013 | 0.65 | 0.009 | 0.48 | 0.005 | 0.28 | 0.004 | 0.21 |
| *Cupriavidus metallidurans* CH34/CCUG 13724 | 0.012 | 0.78 | 0.027 | 1.72 | 0.045 | 2.84 | 0.048 | 3.04 | 0.091 | 5.80 | 0.11 | 6.70 |
| *Cupriavidus necator* ATCC 17699 | 0.019 | 1.23 | 0.029 | 1.90 | 0.043 | 2.85 | 0.054 | 3.60 | 0.090 | 5.99 | 0.15 | 9.91 |
| *Dyadobacter fermentans* DSM 18053 | 0.17 | 8.78 | 0.068 | 3.50 | 0.114 | 5.86 | 0.099 | 5.08 | 0.054 | 2.80 | 0.02 | 1.02 |
| *Pseudomonas stutzeri* JM300/DSM 10701 | 0.022 | 1.42 | 0.025 | 1.58 | 0.045 | 2.82 | 0.064 | 4.05 | 0.129 | 8.16 | 0.13 | 8.46 |
| *Rhodobacter sphaeroides* DSM 158/ATCC 17023 | 0.021 | 1.46 | 0.011 | 0.79 | 0.045 | 3.12 | 0.092 | 6.34 | 0.111 | 7.61 | 0.17 | 11.35 |
| *Salinibacter ruber* DSM 13855 | 0.021 | 1.39 | 0.029 | 1.928 | 0.050 | 3.294 | 0.117 | 7.704 | 0.114 | 7.554 | 0.171 | 11.26 |
| *Sulfurimonas denitrificans* DSM 1251 | 0.26 | 9.85 | 0.287 | 9.889 | 0.123 | 4.260 | 0.043 | 1.475 | 0.002 | 0.073 | 0.008 | 0.28 |
| *Methylosinus trichosporium* Ob3p | 0.001 | 0.07 | 0.005 | 0.343 | 0.003 | 0.188 | 0.003 | 0.206 | 0.006 | 0.387 | 0.003 | 0.21 |
| *Methylocella tundraea* | 0.002 | 0.16 | 0.012 | 0.777 | 0.007 | 0.412 | 0.010 | 0.627 | 0.017 | 1.078 | 0.010 | 0.65 |
| *Methylomicrobium buryatense* 5B | 0.018 | 0.88 | 0.028 | 1.346 | 0.020 | 0.967 | 0.016 | 0.752 | 0.006 | 0.308 | 0.003 | 0.16 |
| *Methanoregula boonei* | 0.001 | 0.028 | 0.001 | 0.059 | 0.002 | 0.103 | 0.001 | 0.050 | 0.019 | 1.039 | 0.001 | 0.06 |
| *Methanolacinia petrolearia* | 0.000 | 0.011 | 0.001 | 0.024 | 0.001 | 0.042 | 0.001 | 0.040 | 0.010 | 0.479 | 0.000 | 0.02 |
| sum: | 1.00 | 47.43 | 1.00 | 48.51 | 1.00 | 52.70 | 1.00 | 56.26 | 1.00 | 59.64 | 1.00 | 62.10 |

Table S3. Soil properties of used samples in the shotgun vs. targeted analysis and targeted vs. archaeal *amoA* amplicon analysis.

| Site | Coordinates | Corg/Norg | pH H_2_O | NH_4_^+^ [μg NH_4_^+^ -N·g -1 dry soil] | NO_2_^-^ [μg NO_2_^-^ -N·g -1 dry soil] | NO_3_^-^ [μg NO_3_^-^ -N·g -1 dry soil] | Fe II [μmol·g -1 dry soil] | Fe III [μmol·g -1 dry soil] | Ref.: |
| --- | --- | --- | --- | --- | --- | --- | --- | --- | --- |
| Agricultural field, Hungary^#~~&~~^ | 47°31N 16°59E | 17.9±5.3 | 6.7±0.16 | 0.02±0.004 | 0.23±0.1 | 3.4±1.9 | 1.5±0.07 | 43.3±4.8 | This study |
| Belfontaine wetland, France^#^ | 46°34N 6°04E | 14±0.6 | 6.9±0.1 | 0.04±0 | 0.04±0 | 0.002±0.001 | 78.5 ± 43.9 | 78.5 ± 43.9 | [22] |

bd = below detection limit

NA = not analyzed

^#^= shotgun vs. targeted comparison, Fig. 2,3.

^&^= Targeted vs. *amoA* amplicon analysis, Fig. 4, S3.

Table S4. Number of total sequences produced, targeted reads and recovery of targeted sequences after capture and depth of the sequencing.

| Sample: | Sequencing method | Total reads | | Targeted reads | % Targeted from total | Depth, of bases: | |
| --- | --- | --- | --- | --- | --- | --- | --- |
| Mock community GC 47% | Targeted metagenomics | 1844716 | 1128755 | | 61.2 | 1.0G |  |
| Mock community GC 50% | Targeted metagenomics | 2281740 | 1437919 | | 63.0 | 1.3G |  |
| Mock community GC 53% | Targeted metagenomics | 2006493 | 1360701 | | 67.8 | 1.1G |  |
| Mock community GC 57% | Targeted metagenomics | 2346193 | 1692037 | | 72.1 | 1.3G |  |
| Mock community GC 60% | Targeted metagenomics | 2625812 | 1771389 | | 67.5 | 1.5G |  |
| Mock community GC 63% | Targeted metagenomics | 2429736 | 1640046 | | 67.5 | 1.3G |  |
| 44 FR_Bellfonte_wetland_BF_3_8 | Targeted metagenomics | 214947 | 124886 | | 58.1 | 114.4M |  |
| 50 FR_Bellfonte_wetland_BF_10-1 | Targeted metagenomics | 285234 | 161399 | | 56.6 | 147.5M |  |
| 51 FR_Bellfonte_wetland_BF_10-2 | Targeted metagenomics | 198756 | 109938 | | 55.3 | 104.1M |  |
| 47 HG_agricultural_soil_H16 | Targeted metagenomics | 246209 | 130449 | | 53.0 | 132M |  |
| 48 HG_agricultural_soil_H17 | Targeted metagenomics | 220005 | 116976 | | 53.2 | 117.5M |  |
| 49 HG_agricultural_soil_H18 | Targeted metagenomics | 311535 | 162396 | | 52.1 | 160.7M |  |
|  |  |  |  | |  |  |  |
| Bellfonte_wetland BF_3-8 | Shotgun metagenomics | 21464245 | 5102 | | 0.024 | 6.4G |  |
| Bellfonte_wetland BF_10-1 | Shotgun metagenomics | 43620482 | 6434 | | 0.015 | 12.9G |  |
| Bellfonte_wetland BF_10-2 | Shotgun metagenomics | 50203331 | 6865 | | 0.014 | 14.9G |  |
| agricultural_soil H16 | Shotgun metagenomics | 53422156 | 3457 | | 0.007 | 16G |  |
| agricultural_soil H17 | Shotgun metagenomics | 22140941 | 2883 | | 0.013 | 6.6G |  |
| agricultural_soil H18 | Shotgun metagenomics | 57814232 | 4213 | | 0.007 | 17.3G |  |
|  |  |  |  | |  |  |  |
| Agricultural H16 | Amplicon sequencing | 58006 | 49679 | | 85.64 | 31M |  |
| Agricultural H17 | Amplicon sequencing | 27565 | 23648 | | 85.79 | 14.9M |  |
| Agricultural H18 | Amplicon sequencing | 35955 | 30830 | | 85.75 | 19.3M |  |

Fig. S3. Phylogenetic placement of *nosZ* reads obtained from agricultural soil using (n=3) A) shotgun metagenomics and B) targeted metagenomics (n=3). For illustration purposes all the replicas are pooled together. The reference phylogeny[12] is based on amino acid sequences analyzed using the LG+R10 substitution model in IQ-TREE, and node symbols indicate the location of placements in the reference tree. Symbol size corresponds to the relative abundance of reads placed at each node, and the scale bar indicates branch length in the reference tree. ‘Outgroup’ denotes distant homologues of *nosZ* with unknown function.


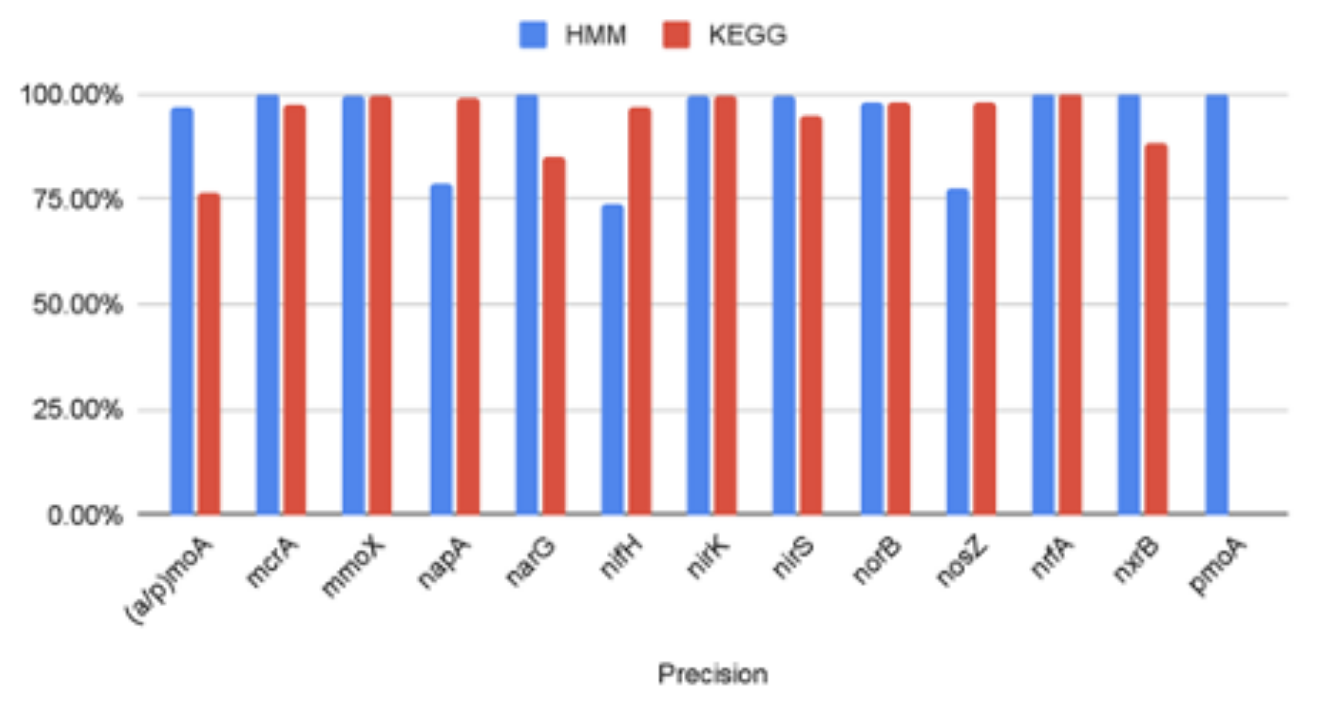

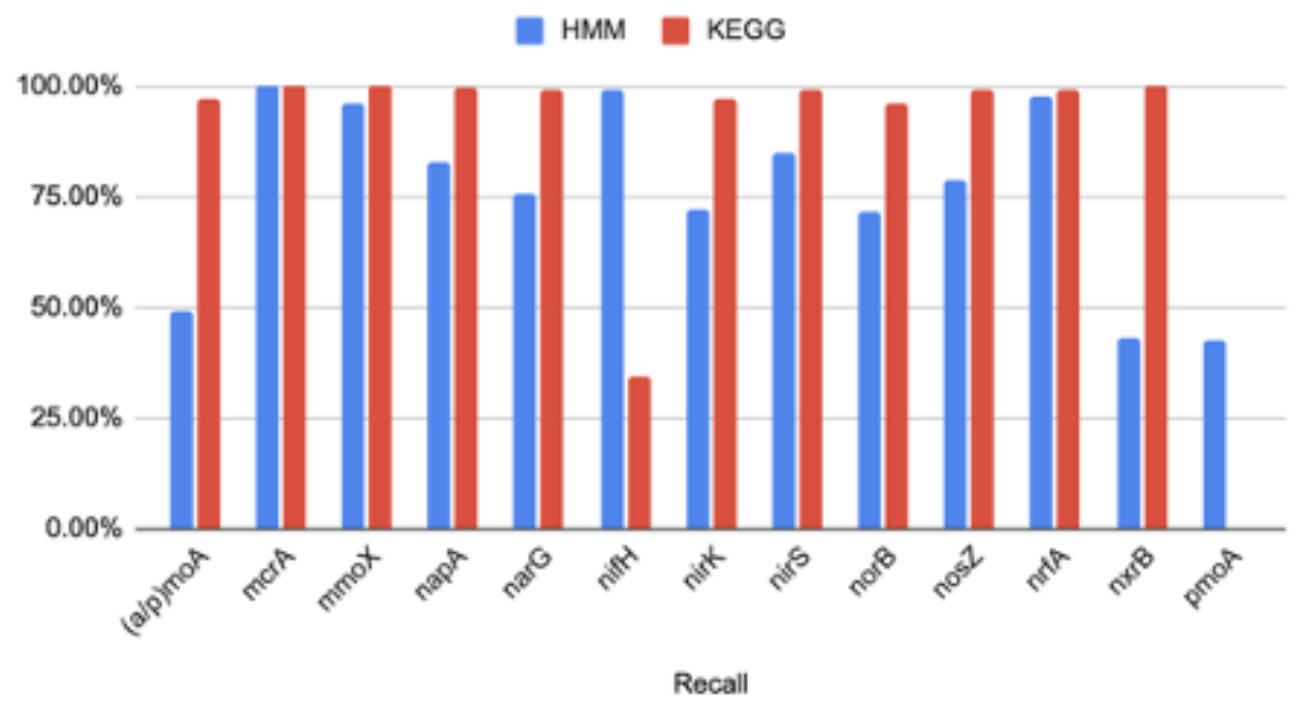
Fig. S4. Comparison of KEGG annotation and custom HMMs precision and recall for the mock-community comparison with different GC% categories combined.


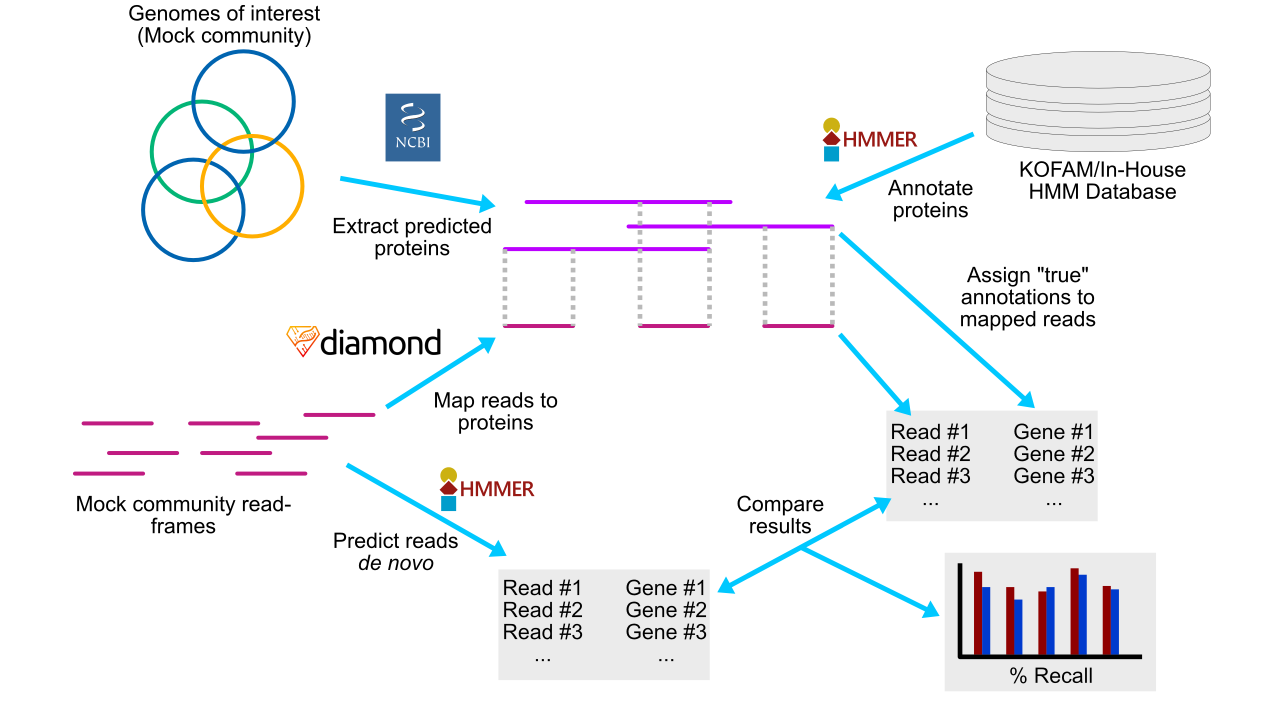


Fig. S5. Illustration of pipeline for determining TP, FP, TN, and FN de novo predictions by read mapping and HMM model prediction compared to genome predicted proteins.

**References**

1. Manoharan, L., Kushwaha, S. K., Hedlund, K., Ahrén, D. Captured metagenomics: large-scale targeting of genes based on ‘sequence capture’ reveals functional diversity in soils. *DNA Res.* 2015; 22: 451-460.

2. Hyatt, D., Chen, G.L., LoCascio, R.F. *et al.* Prodigal: prokaryotic gene recognition and translation initiation site identification. *BMC Bioinformatics* 2010; 11: 119 <https://doi.org/10.1186/1471-2105-11-119>

3. Aramaki, T., Blanc-Mathieu, R., Endo, H., Ohkubo, K., Kanehisa, M., Goto, S., Ogata, H. KofamKOALA: KEGG Ortholog assignment based on profile HMM and adaptive score threshold. *Bioinform.* 2020; 36:2251-2252. Doi: 10.1093/bioinformatics/btz859

4. Eddy S.R. Accelerated profile HMM searches. PLOS Comp. Biol. 2011; 7:e1002195, 2011

5. Bagnoud, A., Siljanen, H. Zenodo: alex-bagnoud/probe-capture: v2025-06. 2025; <https://doi.org/10.5281/zenodo.15752134>

6. Alves, R.J.E., Minh, B.Q., Urich, T., von Haeseler, A., Schleper, C. Unifying the global phylogeny and environmental distribution of ammonia-oxidizing archaea based on amoA genes. *Nat. Comm.* 2018; 9: 1517. <https://doi.org/10.1038/s41467-018-03861-1>.

7. Edgar, R.C. Search and clustering orders of magnitude faster than BLAST. *BMC Bioinformatics* 2010; 26: 2460–2461

8. Edgar, R.C., Haas, B.J., Clemente, J.C., Quince, C., Knight, R. UCHIME improves sensitivity and speed of chimera detection. *BMC Bioinformatics* 2011; 27: 2194–2200.

9. Caporaso, J.G., Kuczynski, J., Stombaugh, J., Bittinger, K., Bushman, F.D., Costello, E.K., Fierer, N., Gonzalez Peña, A., Goodrich, J.K., Gordon, J.I., Huttley, G.A., Kelley, S.T., Knights, D., Koenig, J.E., Ley, R.E., Lozupone, C.A., McDonald, D., Muegge, B.D., Pirrung, M., Reeder, J., Sevinsky, J.R., Turnbaugh, P.J., Walters, W.A., Widmann, J., Yatsunenko, T., Zeneveld, J., Knight, R. QIIME allows analysis of Highthroughput community sequencing data. *Nat. Methods* 2010; 7: 335–336.

10. Altschul, S. F., Gish, W., Miller, W., Myers, E. W., Lipman, D. J. Basic local alignment search tool. *J. Mol. Biol.* 1990; 215: 403-410.

11. Callahan, B.J., McMurde, P.J., Rosen, M.J., Han, A.W., Johnson, A.J., Holmes, S.P. DADA2: high-resolution sample inference from Illumina amplicon data. *Nat. Methods* 2016; 13: 581–583.<https://doi.org/10.1038/nmeth.3869>.

12. Graf, D.R.H., Jones, C.M., Zhao, M., Hallin, S. Assembly of root-associated N2O-reducing communities of annual crops is governed by selection for nosZ clade I over clade II. *FEMS Microbiol. Ecol.* 2022; 98:1-11

13. Price, M.N., Dehal, P.S., Arkin, A.P. FastTree 2--approximately maximum-likelihood trees for large alignments. *PLoS ONE* 2010; 5: e9490.

14. Trifinopoulos, J., Nguyen, L.T., von Haeseler, A., Minh, B.Q. W-IQ-TREE: a fast online phylogenetic tool for maximum likelihood analysis. *Nucleic Acids Res.* 2016; 44(W1):W232-5. doi: 10.1093/nar/gkw256.

15. Fu, L., Niu, B. Zhu, Z., Wu, S., Li, W. CD-HIT: accelerated for clustering the next-generation sequencing data. *Bioinformatics*. 2012; 28: 3150–3152. <https://doi.org/10.1093/bioinformatics/bts565>

16. Barbera, P., Kozlov, A.M., Czech, L., Morel, B., Darriba, D., Flouri, T., Stamatakis, A. EPA-ng: Massively Parallel Evolutionary Placement of Genetic Sequences. *Syst. Biol.* 2019; 68: 365-369.

17. Yu, G.C., Smith, D.K., Zhu, H.C., Guan, Y., Lam, T.T.Y. GGTREE: an R package for visualization and annotation of phylogenetic trees with their covariates and other associated data. *Methods in Ecol Evol.* 2017; 8: 28-36.

18. Cerqueira, N.M.F.S.A., Gonzalez, P.J., Fernandes, P.A., Moura, J.J.G., Ramos, M.A. [Periplasmic Nitrate Reductase and Formate Dehydrogenase : Similar Molecular Architectures with Very Different Enzymatic Activities.](https://pubs.acs.org/doi/10.1021/acs.accounts.5b00333) *Acc. Chem. Res.* 2015; 48: 2875-2884 DOI: 10.1021/acs.accounts.5b00333

19. Putkinen, A., Siljanen, H.M.P., Laihonen, A., Paasisalo, I., Porkka, K., Tiirola, M., Haikarainen, I., Tenhovirta, S. and Pihlatie, M. New insight to the role of microbes in the methane exchange in trees: evidence from metagenomic sequencing. *New Phytol* 2021; 231: 524-536.<https://doi.org/10.1111/nph.17365>

20. Glaze, T.D., Erler, D.V., Siljanen, H.M.P. Microbially facilitated nitrogen cycling in tropical corals. *ISME J.* 2021; 16: 68–77 doi: 10.1038/s41396-021-01038-1.

21. Marushchak ME, Kerttula J, Diáková K. *et al.* Thawing Yedoma permafrost is a neglected nitrous oxide source. *Nat Commun* 2021: 12: 7107. <https://doi.org/10.1038/s41467-021-27386-2>

22. Bagnoud, A., Guye-Humbert, S., Schloter-Hai, B., Schloter, M. Zopfi, J. Environmental factors determining distribution and activity of anammox bacteria in minerotrophic fen soils, FEMS Microb Ecol 2020; 96: fiz191, <https://doi.org/10.1093/femsec/fiz191>
